# Lymphopenia associated with sphingosine 1-phosphate receptor modulators (S1PRMs) in multiple sclerosis: analysis of European pharmacovigilance data

**DOI:** 10.1007/s43440-025-00725-6

**Published:** 2025-04-09

**Authors:** Nunzia Balzano, Raffaella Di Napoli, Federica Fraenza, Daniele Di Giulio Cesare, Ornella Moreggia, Mirko Cardillo, Cristina Scavone, Giorgia Teresa Maniscalco, Annalisa Capuano, Liberata Sportiello

**Affiliations:** 1 Campania Regional Centre for Pharmacovigilance and Pharmacoepidemiology, University of Campania “Luigi Vanvitelli”, Naples, 80138 Italy; 2 Department of Experimental Medicine, University of Campania “Luigi Vanvitelli”, Naples, 80138 Italy; 3 Multiple Sclerosis Regional Center, “A. Cardarelli” Hospital, Naples, 80131 Italy; 4 Department of Life Science, Health, and Health Professions, Link Campus University, Roma, Italy; 5 Neurological Clinic and Stroke Unit, “A. Cardarelli” Hospital, Naples, 80131 Italy

**Keywords:** Lymphopenia, Sphingosine 1-Phosphate receptor modulators (S1PRMs), Multiple sclerosis, Spontaneous adverse event reporting, Pharmacovigilance database

## Abstract

**Background:**

The treatment landscape for Multiple Sclerosis (MS) has increased significantly over the past few decades, thanks to the introduction of disease-modifying therapies (DMTs). Fingolimod, siponimod, ozanimod, and ponesimod belong to the newer generation of oral DMTs categorized as sphingosine 1-phosphate receptor modulators (S1PRMs). Because of their mechanism of action, they may increase the risk of lymphopenia, which could influence the therapeutic management of people with MS. The aim of this study was to describe and compare the reporting frequency of lymphopenia related to four S1PRMs.

**Methods:**

Individual case safety reports (ICSRs) were retrieved from the European spontaneous reporting system database (EudraVigilance) from January 1st, 2022, to December 31st, 2023. The reporting odds ratios (RORs) were computed to compare the reporting probability of lymphopenia between a S1PRM versus each other.

**Results:**

We retrieved 4017 ICSRs, of which 521 (13%) reported lymphopenia associated with fingolimod (53.3%), siponimod (38.4%), ozanimod (5.4%), and ponesimod (2.1%). The most common reporting source was the healthcare professional (94.2%), and more than half of the ICSRs (62.6%) reported serious lymphopenia. Fingolimod was associated with a lower reporting frequency of lymphopenia compared to siponimod. Both siponimod and fingolimod were associated with a higher reporting frequency of lymphopenia compared to ozanimod; siponimod also had a higher reporting probability in comparison with ponesimod.

**Conclusions:**

The most relevant clinical implication of the disproportionality analysis is to increase the awareness of the risk of lymphopenia related to these drugs, thus supporting proactive monitoring and optimizing treatment strategies for people with MS.

**Clinical trial number:**

Not applicable.

**Supplementary Information:**

The online version contains supplementary material available at 10.1007/s43440-025-00725-6.

## Introduction

Multiple sclerosis (MS) is a chronic autoimmune disease of the central nervous system characterized by inflammation, demyelination, and neurodegeneration. It can present with a wide range of symptoms, including fatigue, limb weakness, optic neuritis, balance and coordination problems, muscle spasms and stiffness, cognitive impairment, pain, and speech and swallowing difficulties [1–3]. Whilst most diagnoses occur between the ages of 20 and 50, MS can manifest at any point in life [4]. The Multiple Sclerosis International Federation (MSIF) has estimated a rise in the global MS population, with numbers climbing from 2.3 million in 2013 to 2.8 million in 2020 and further to 2.9 million in 2023 [5]. It is widely recognized that the costs associated with MS are significant and tend to increase in parallel with the progression of the patient’s disability. In Europe, the average annual cost per patient has been estimated at €22,800 for mild cases, €37,100 for moderate cases, and €57,500 for severe cases [6]. MS can present with different clinical courses, with relapsing-remitting MS (RRMS) being the most common form, characterized by periods of relapses followed by partial or complete recovery [1]. Over time, some individuals with RRMS transition to secondary progressive MS (SPMS), marked by a steady progression of disability with or without superimposed relapses. Compared to RRMS, SPMS generally affects an older population [7]. The treatment landscape for MS has expanded significantly over the past few decades, largely due to the introduction of disease-modifying therapies (DMTs). These drugs are specifically crafted to reduce disease activity, which refers to decreasing the inflammation and neurological damage associated with MS. This includes reducing relapses, slowing the progression of the disease, and improving the quality of life for individuals living with this condition [8, 9]. Fingolimod, siponimod, ozanimod, and ponesimod are oral DMTs categorized as sphingosine 1-phosphate receptor modulators (S1PRMs). Fingolimod was the first S1PRM approved on 17 March 2011 for adults with RRMS/highly active RRMS or cases active despite another DMT, and it is currently the only one approved also for adolescents and children [10]. Siponimod is more selective and is the only S1PRM approved for active SPMS, which is defined by the presence of relapses or inflammatory activity on imaging. It received marketing authorization on 13 January 2020 [11]. The most recent S1PRM available for treating relapsing-remitting forms of MS are ozanimod and ponesimod, which were approved on 20 May 2020 and 19 May 2021, respectively [12, 13]. These drugs act by modulating S1P receptors, leading to their internalization and functional antagonism. This prevents the egress of lymphocytes from lymph nodes, trapping them in a non-proliferative state and thereby reducing their infiltration into the central nervous system. As a result, S1P receptor modulators decrease the autoimmune-mediated attack on myelin, mitigate inflammation associated with MS, and may help reduce further neurodegeneration [14]. Since their marketing introduction, S1PRMs have been associated with the onset of several adverse effects. According to their European Union Risk Management Plans, collective important identified and potential risks are bradyarrhythmia (including conduction defects and bradycardia complicated by hypotension) occurring post-first dose, liver transaminase elevation, macular edema, skin cancers (Basal cell carcinoma, Kaposi’s sarcoma, Malignant melanoma, Merkel cell carcinoma, Squamous cell carcinoma), convulsions, lymphoma and opportunistic infections including progressive multifocal leukoencephalopathy (PML), Varicella Zoster virus (VZV), herpes viral infections other than VZV, fungal infection [15–18]. Moreover, because of their mechanism of action, the S1PRMs may increase the risk of lymphopenia [14, 19]. Thus, a complete blood count should be performed before initiating a S1PRM and periodically thereafter. Given the potential for liver function test (LFT) abnormalities, regular monitoring of LFTs is also suggested. The European Medicines Agency (EMA) has indeed recommended interrupting treatments if the lymphocyte count drops below 0.2 × 10^9/L [10–13] or reducing the dose with subsequent reassessment. Some real-world studies suggest that a majority of people with MS treated with S1PRMs exhibit grade 2–4 lymphopenia shortly after initiating therapy with a dose-dependent reduction in total peripheral lymphocytes [20–23]. Considering the interest of neurologists for understanding more details on this safety concern for a better therapeutic management of people with MS, we choose to conduct a pharmacovigilance study to examine the reporting frequency of lymphopenia by comparing each S1PRM authorized to date (fingolimod, siponimod, ozanimod, and ponesimod) by using data collected in the European spontaneous reporting system database. We hypothesize that the reporting frequency of lymphopenia will differ between the four S1PRMs and aim to assess whether the use of different S1PRMs in people with MS is associated with an increased reporting frequency of lymphopenia.

## Methods

### Study design

We conducted a database-related pharmacovigilance study to analyse the reporting frequency of lymphopenia associated with S1PRMs through a disproportionality analysis. The REporting of A Disproportionality analysis for drug Safety signal detection using ICSRs in PharmacoVigilance (READUS-PV) guideline supported our reporting of the results of the disproportionality analyses(Supplementary Table 1) [24].

### Data source

In the current stud, we utilized the European pharmacovigilance database (Eudravigilance, EV) owned by the EMA. Data are accessible to the public via the EMA website (www.adrreports.eu). EV database contains Individual Case Safety Reports (ICSRs) of suspected adverse drug reactions (ADRs) and adverse events following immunization (AEFI) related to authorized medications or vaccines respectively. All ICSRs, submitted by healthcare professionals, patients, and citizens, can be downloaded in a single Excel file. Each ICSR included patient age group, sex, type of reporting (spontaneous or not spontaneous), primary source qualification (Healthcare Professional, Non-Healthcare Professional), geographic origin (European Economic Area, Non-European Economic Area), adverse reaction list, suspect/interacting drug list, concomitant drug list, outcome (recovered/resolved, not recovered/not resolved, recovering/resolving, recovered/resolved with sequelae, fatal, unknown) and seriousness (caused/prolonged hospitalisation, other medically important condition, life threatening, congenital anomaly, disabling, results in death). The Adverse Reaction List is coded with the Medical Dictionary for Regulatory Activities (MedDRA), which is a standardized medical terminology globally used for adverse events classification. MedDRA is organized into five hierarchical levels, ranging from the most detailed to the most generic: lowest level terms (LLT), preferred terms (PT), high-level terms (HLT), high-level group terms (HLGT), and system organ class (SOC). By analysing data from the EV database, we were able to uncover safety information from real-world settings and identify new insights into drug safety that may not have been apparent during pre-marketing clinical trials.

### ICSRs selection

We retrieved all the ICSRs related to each S1PRM (fingolimod, ozanimod, siponimod, and ponesimod) from January 1st, 2022, to December 31st, 2024, using the line-listing function on the EV website. Firstly, we conducted a preliminary selection of Excel files by searching for PTs “lymphopenia” and/or “lymphocyte count decreased” and/or “T-lymphocyte count decreased” and/or “B-lymphocyte count decreased” in the column of the PT list. Next, we applied exclusion criteria to refine the dataset further. Specifically, we removed the ICSRs where ozanimod was listed as a suspected drug but was associated with “ulcerative colitis” or had no reported therapeutic indication and off-label cases for fingolimod and siponimod with no explicated therapeutic indication. Subsequently, we identified potential duplicates by comparing case IDs and removed them from the dataset.

### Descriptive analysis

For each S1PRM, we registered sex (male or female) and age group, reporter type (Healthcare Professionals or Non-Healthcare Professionals), country (European Economic Area or Non-European Economic Area), adverse events (type, seriousness, and outcome) and treatments (other suspected drugs and concomitant drugs). All ADRs were categorized according to the MedDRA. The seriousness of ADRs was assessed in accordance with the International Council on Harmonization E2D guidelines. Specifically, an ADR was classified as “serious” if it resulted in death, life-threatening, required or prolonged hospitalization, caused persistent or significant disability/incapacity, resulted in a congenital anomaly/congenital disability, or led to other clinically important conditions. When additional criteria were provided for each ADR, we selected the most serious criterion for classification. Moreover, the outcome of ADRs was considered favourable if it resulted in “recovered/resolved” or “recovering/resolving”. Conversely, the outcome was classified as unfavourable if it resulted in “recovered/resolved with sequelae”, “not recovered/not resolved”, or “fatal”. All qualitative variables were expressed as numbers and percentages.

### Disproportionality analysis

We performed a disproportionality analysis to assess the reporting frequency of lymphopenia for fingolimod, siponimod, ozanimod, and ponesimod compared to each other, applying the Reporting Odds Ratio (ROR) with its 95% confidence interval (95% CI). Moreover, the ROR was also computed for each S1PRM (fingolimod, siponimod, ozanimod, and ponesimod) compared to the combination fingolimod/siponimod. The ROR was calculated as (a/c)/(b/d): “a” is the number of events reported with the drug of interest, “c” is the number of events reported with the comparator, “b” is the number of other events reported with the drug of interest, and “d” is the number of other events reported with the comparator. At least 3 events were required for each drug to perform disproportionality analyses, and a p-value ≤ 0.05 was applied for statistical significance. Data management and analyses were performed using Excel 365 (Microsoft Office) and R (version 4.2.2, R Development Core Team).

## Results

**Fig. 1:**
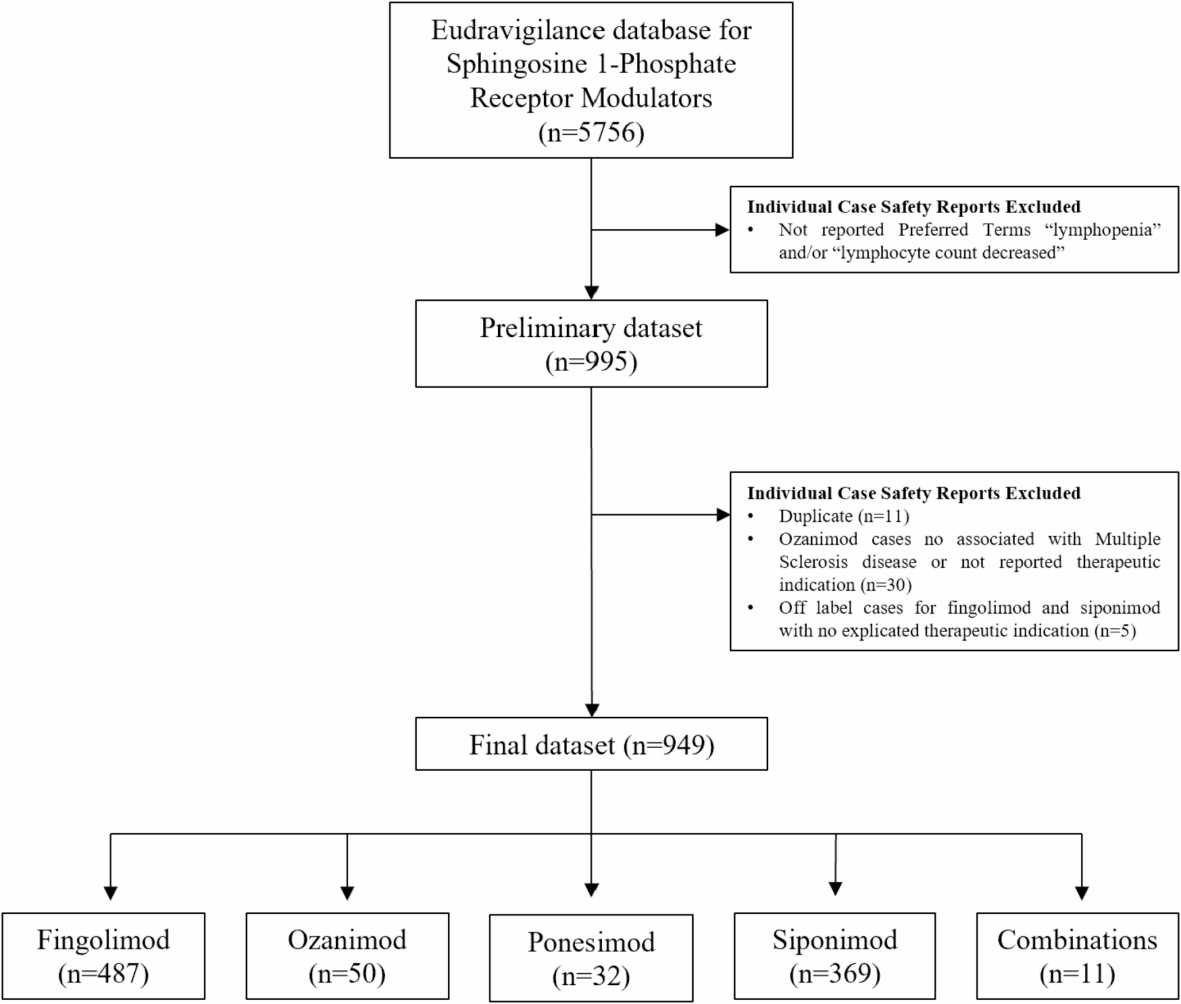
Flowchart of the selection process of Individual Case Safety Reports (ICSRs) from the EudraVigilance database

During the study period, we retrieved a total of 5756 ICSRs from the EV database. Of these, 949 cases met the selection criteria, of which 487 (51.3%) related to fingolimod, 369 (38.9%) to siponimod, 50 (5.3%) to ozanimod, 32 (3.4%) to ponesimod, and 11 (1.2%) to a combination of S1PRMs. The selection process of ICSRs from the EV database is shown in the flowchart (Fig. 1).

Overall, the majority of the ICSRs referred to adults, and the age group most represented was 18–64 years (*N* = 696; 73.3%). The female sex was reported for 695/949 (73.2%) of cases. All ICSRs were spontaneous (*N* = 949, 100.0%), and the most common reporting source was the healthcare professional (*N* = 884; 93.2%). The primary source country for regulatory purposes of ICSRs was the European Economic Area (*N* = 649; 68.4%). In 868/949 (91.5%), an S1PRM was the only suspected drug to have caused the adverse event. “Multiple sclerosis” was the most reported therapeutic indication (*N* = 605; 63.8%), followed by “Secondary progressive multiple sclerosis” (*N* = 175; 18.4%), “Relapsing-remitting multiple sclerosis” (*N* = 161; 17,0%), and “Progressive multiple sclerosis” (*N* = 6; 0.6%). Moreover, no concomitant medication was reported in 80.1% (*N* = 760) of ICSRs. The distribution of concomitant drugs listed in the remaining 19.9% of cases is shown in Supplementary Table 2. All characteristics of ICSRs for each S1PRM are presented in Table 1.

**Table 1:** Characteristics of individual case safety reports (ICSRs) reporting lymphopenia with sphingosine 1-phosphate receptor modulators recognized in the eudravigilance spontaneous reporting system from 1st January 2022 to 31st December 2024

	Fingolimod (*N* = 487)	Ozanimod (*N* = 50)	Ponesimod (*N* = 32)	Siponimod (*N* = 369)	Fingolimod/Siponimod (*N* = 10)	Siponimod/Ozanimod (*N* = 1)	Overall (*N* = 949)
**Age**							
0–1 Month	3 (0.6)	-	-	-	-	-	3 (0.3)
3–11 Years	1 (0.2)	-	-	-	-	-	1 (0.1)
12–17 Years	16 (3.3)	-	-	-	-	-	16 (1.7)
18–64 Years	388 (79.7)	35 (70.0)	16 (50.0)	250 (67.8)	7 (70.0)	-	696 (73.3)
65–85 Years	15 (3.1)	3 (6.0)	1 (3.1)	18 (4.9)	-	1 (100)	38 (4.0)
Not Specified	64 (13.1)	12 (24.0)	15 (46.9)	101 (27.4)	3 (30.0)	-	195 (20.5)
**Sex**							
Female	366 (75.2)	39 (78.0)	25 (78.1)	258 (69.9)	6 (60.0)	1 (100)	695 (73.2)
Male	111 (22.8)	10 (20.0)	7 (21.9)	95 (25.7)	4 (40.0)	-	227 (23.9)
Not Specified	10 (2.1)	1 (2.0)	-	16 (4.3)	-	-	27 (2.8)
**Source**							
Healthcare Professional	456 (93.6)	45 (90.0)	29 (90.6)	344 (93.2)	9 (90.0)	1 (100)	884 (93.2)
Non Healthcare Professional	31 (6.4)	5 (10.0)	3 (9.4)	25 (6.8)	1 (10.0)	-	65 (6.8)
**Country**							
European Economic Area	298 (61.2)	46 (92.0)	31 (96.9)	270 (73.2)	3 (30.0)	1 (100)	649 (68.4)
Non-European Economic Area	189 (38.8)	4 (8.0)	1 (3.1)	99 (26.8)	7 (70.0)	-	300 (31.6)
**Indication**							
Foetal exposure during pregnancy	3 (0.6)	-	-	-	-	-	3 (0.3)
Multiple sclerosis	336 (69.0)	36 (72.0)	29 (90.6)	196 (53.1)	6 (60.0)	1 (100)	604 (63.6)
Relapsing-remitting multiple sclerosis	134 (27.5)	14 (28.0)	3 (9.4)	9 (2.4)	1 (10.0)	-	161 (17.0)
Secondary progressive multiple sclerosis	14 (2.9)	-	-	158 (42.8)	3 (30.0)	-	175 (18.4)
Progressive multiple sclerosis	-	-	-	6 (1.6)	-	-	6 (0.6)
**Suspects**							
1	439 (90.1)	49 (98.0)	32 (100)	348 (94.3)	-	-	868 (91.5)
2	36 (7.4)	-	-	10 (2.7)	8 (80.0)	1 (100)	55 (5.8)
3	8 (1.6)	-	-	1 (0.3)	-	-	9 (0.9)
4	3 (0.6)	1 (2.0)	-	-	2 (20.0)	-	6 (0.6)
≥ 5	1 (0.2)	-	-	10 (2.7)	-	-	11 (1.2)
**Concomitants**							
1	35 (7.2)	9 (18.0)	1 (3.1)	18 (4.9)	-	-	63 (6.6)
2	14 (2.9)	2 (4.0)	1 (3.1)	14 (3.8)	-	1 (100)	32 (3.4)
3	13 (2.7)	1 (2.0)	3 (9.4)	16 (4.3)	1 (10.0)	-	34 (3.6)
4	4 (0.8)	-	-	7 (1.9)	-	-	11 (1.2)
≥ 5	25 (5.1)	2 (4.0)	1 (3.1)	21 (5.7)	-	-	49 (5.2)
Not reported	396 (81.3)	36 (72.0)	26 (81.3)	293 (79.4)	9 (90.0)	-	760 (80.1)

Data are expressed as N (%)

We observed a total of 949 cases of lymphopenia events. The majority of lymphopenia events associated with fingolimod (*N* = 297; 61.0%) and siponimod (*N* = 237; 64.2%) were serious, whereas those related to ozanimod (*N* = 39; 78.0%) and ponesimod (*N* = 23; 71.9%) were not serious. The most reported seriousness criterion was other medically important conditions for both fingolimod (*N* = 254; 52.2%) and siponimod (*N* = 214; 58.0%,). The outcome was unknown for 456 events. There were only two fatal cases (0.2%). The seriousness and outcomes criteria are shown in Table 2.

**Table 2:** Seriousness and outcome of lymphopenia for sphingosine 1-phosphate receptor modulators recognized in the eudravigilance spontaneous reporting system from 1st January 2022 to 31st December 2024

	Fingolimod (*N* = 487)	Ozanimod (*N* = 50)	Ponesimod (*N* = 32)	Siponimod (*N* = 369)	Fingolimod/Siponimod (*N* = 10)	Siponimod/Ozanimod (*N* = 1)	Overall (*N* = 949)
**Seriousness**							
Not serious	190 (39.0)	39 (78.0)	23 (71.9)	132 (35.8)	3 (30.0)	1 (100)	388 (40.9)
Results in Death	1 (0.2)	-	-	1 (0.3)	-	-	2 (0.2)
Life Threatening	14 (2.9)	-	-	5 (1.4)	-	-	19 (2.0)
Caused/Prolonged Hospitalisation	28 (5.7)	1 (2.0)	-	17 (4.6)	-	-	46 (4.8)
Other Medically Important Condition	254 (52.2)	10 (20.0)	9 (28.1)	214 (58.0)	7 (70.0)	-	494 (52.1)
**Outcome**							
Fatal	1 (0.2)	-	-	1 (0.3)	-	-	2 (0.2)
Not Recovered/Not Resolved	74 (15.2)	10 (20.0)	3 (9.4)	68 (18.4)	-	-	155 (16.3)
Recovered/Resolved With Sequelae	-	-	-	2 (0.5)	-	-	2 (0.2)
Recovering/Resolving	66 (13.6)	6 (12.0)	1 (3.1)	50 (13.6)	-	-	123 (13.0)
Recovered/Resolved	120 (24.6)	7 (14.0)	8 (25.0)	73 (19.8)	3 (30.0)	-	211 (22.2)
Unknown	226 (46.4)	27 (54.0)	20 (62.5)	175 (47.4)	7 (70.0)	1 (100)	456 (48.1)

Data are expressed as N (%)

Moreover, 59.7% of ICSRs (*N* = 567) reported at least one adverse event other than lymphopenia, for a total of 1908 other adverse events. The distribution of specific all events is listed in Supplementary Table 3. Categorizing all other events by MedDRA SOCs (Table 3), the highest percentage emerged for the “Nervous system disorder” SOC (*N* = 471; 24.7%), followed by “Investigation” (*N* = 284; 14.9), and “Infection and infestations” (*N* = 220; 11.5%). As shown in Table 4, the most common HLGT within the “Nervous system disorders” SOC was “Neurological disorders NEC”, while for the “Investigations” SOC, it was “Haematology investigations (including blood groups)”. Within the “Infections and infestations” SOC, the most frequently reported HLGT was “Viral infectious disorders”, with COVID-19 (*N* = 27; 28.1%), PML (*N* = 19; 19.8%), and Herpes zoster (*N* = 14; 14.6%) as the most represented PTs. A complete list of HLGTs for the three most-reported SOCs is provided in Supplementary Tables 4–6.

**Table 3:** Distribution of other adverse events categorized by MedDRA system organ class (SOC) and reported in individual case safety reports (ICSRs) with sphingosine 1-phosphate receptor modulators recognized in the eudravigilance spontaneous reporting system from 1st January 2022 to 31st December 2024

	Fingolimod (*N* = 1152)	Ozanimod (*N* = 54)	Ponesimod (*N* = 30)	Siponimod (*N* = 640)	Fingolimod/Siponimod (*N* = 32)	Overall (*N* = 1908)
**SOC**						
Blood and lymphatic system disorders	52 (4.5)	7 (13.0)	4 (13.3)	23 (3.6)	-	86 (4.5)
Cardiac disorders	17 (1.5)	1 (1.9)	1 (3.3)	12 (1.9)	3 (9.4)	34 (1.8)
Congenital, familial, and genetic disorders	2 (0.2)	-	-	-	-	2 (0.1)
Ear and labyrinth disorders	2 (0.2)	-	-	1 (0.2)	-	3 (0.2)
Endocrine disorders	2 (0.2)	-	-	-	-	2 (0.1)
Eye disorders	37 (3.2)	-	-	10 (1.6)	-	47 (2.5)
Gastrointestinal disorders	50 (4.3)	1 (1.9)	1 (3.3)	27 (4.2)	1 (3.1)	80 (4.2)
General disorders and administration site conditions	122 (10.6)	5 (9.3)	4 (13.3)	73 (11.4)	5 (15.6)	209 (11.0)
Hepatobiliary disorders	17 (1.5)	-	-	8 (1.3)	1 (3.1)	26 (1.4)
Immune system disorders	18 (1.6)	3 (5.6)	-	1 (0.2)	-	22 (1.2)
Infections and infestations	145 (12.6)	11 (20.4)	2 (6.7)	61 (9.5)	1 (3.1)	220 (11.5)
Injury, poisoning, and procedural complications	69 (6.0)	3 (5.6)	1 (3.3)	28 (4.4)	2 (6.3)	103 (5.4)
Investigations	150 (13.0)	5 (9.3)	7 (23.3)	119 (18.6)	3 (9.4)	284 (14.9)
Metabolism and nutrition disorders	12 (1.0)	-	-	1 (0.2)	-	13 (0.7)
Musculoskeletal and connective tissue disorders	29 (2.5)	-	1 (3.3)	41 (6.4)	1 (3.1)	72 (3.8)
Neoplasms benign, malignant and unspecified (incl cysts and polyps)	27 (2.3)	1 (1.9)	-	8 (1.3)	-	36 (1.9)
Nervous system disorders	305 (26.5)	9 (16.7)	2 (6.7)	140 (21.9)	15 (46.9)	471 (24.7)
Pregnancy, puerperium, and perinatal conditions	2 (0.2)	-	-	-	-	2 (0.1)
Product issues	1 (0.1)	-	-	1 (0.2)	-	2 (0.1)
Psychiatric disorders	27 (2.3)	5 (9.3)	-	15 (2.3)	-	47 (2.5)
Renal and urinary disorders	21 (1.8)	-	-	37 (5.8)	-	58 (3.0)
Reproductive system and breast disorders	4 (0.3)	-	-	1 (0.2)	-	5 (0.3)
Respiratory, thoracic, and mediastinal disorders	10 (0.9)	2 (3.7)	1 (3.3)	19 (3.0)	-	32 (1.7)
Skin and subcutaneous tissue disorders	17 (1.5)	-	6 (20.0)	7 (1.1)	-	30 (1.6)
Social circumstances	-	-	-	1 (0.2)	-	1 (0.1)
Surgical and medical procedures	3 (0.3)	-	-	-	-	3 (0.2)
Vascular disorders	11 (1.0)	1 (1.9)	-	6 (0.9)	-	18 (0.9)

Data are expressed as N (%)

**Table 4:** Distribution of the most reported preferred terms (PTs) within the most reported High-Level group terms (HLGTs) of the three most reported system organ classes (SOCs) in individual case safety reports (ICSRs) with sphingosine 1-phosphate receptor modulators recognized in the eudravigilance spontaneous reporting system from 1st January 2022 to 31st December 2024

	Fingolimod	Ozanimod	Ponesimod	Siponimod	Fingolimod/Siponimod	Overall
**Nervous system disorders**						
Neurological disorders NEC	126 (41.3)	1 (11.1)	0 (0)	36 (25.7)	4 (26.7)	167 (35.5)
• Hypoaesthesia	19 (15.1)	-	-	1 (2.8)	1 (25.0)	21 (12.6)
• Dysarthria	8 (6.3)	-	-	6 (16.7)	-	14 (8.4)
• Paraesthesia	13 (10.3)			1 (2.8)		14 (8.4)
**Demyelinating disorders**	**86 (28.2)**	**1 (11.1)**	**1 (50.0)**	**27 (19.3)**	**4 (26.7)**	**119 (25.3)**
• Multiple sclerosis relapse	71 (82.6)	-	1 (100)	24 (88.9)	3 (75.0)	63 (52.9)
• Secondary progressive multiple sclerosis	5 (5.8)	-	-	2 (7.4)	1 (25.0)	8 (6.7)
• Relapsing-remitting multiple sclerosis	3 (3.5)	-	-	1 (3.7)	-	4 (3.4)
**Movement disorders (incl parkinsonism)**	**25 (8.2)**	**0 (0)**	**0 (0)**	**31 (22.1)**	**1 (6.7)**	**57 (12.1)**
• Hemiparesis	5 (20.0)	-	-	4 (12.9)	1 (100)	10 (17.5)
• Gait spastic	-	-	-	-	8 (25.8)	8 (14.0)
• Hypokinesia	-	-	-	-	7 (22.6)	7 (12.3)
**Investigations**						
**Haematology investigations (incl blood groups)**	**52 (34.7)**	**1 (20.0)**	**2 (28.6)**	**59 (49.6)**	**0 (0)**	**114 (40.1)**
• White blood cell count decreased	23 (44.2)	-	1 (50.0)	29 (49.2)	-	53 (46.5)
• Neutrophil count decreased	2 (3.8)	1 (100)		4 (6.8)	-	7 (6.1)
• Monocyte count increased	-	-	1 (50.0)	5 (8.5)	-	6 (5.3)
**Hepatobiliary investigations**	**50 (33.3)**	**4 (80.0)**	**5 (71.4)**	**35 (29.4)**	**0 (0)**	**94 (33.1)**
• Gamma-glutamyltransferase increased	12 (24.0)	1 (25.0)	1 (20.0)	10 (28.6)	-	24 (25.5)
• Alanine aminotransferase increased	12 (24.0)	-	1 (20.0)	5 (14.3)	-	18 (19.1)
• Hepatic enzyme increased	9 (18.0)	1 (25.0)	2 (40.0)	5 (14.3)	-	17 (18.1)
**Cardiac and vascular investigations (excl enzyme tests)**	**5 (3.3)**	**0 (0)**	**0 (0)**	**6 (5.0)**	**0 (0)**	**11 (3.9)**
• Blood pressure increased	1 (20.0)	-	-	2 (33.3)	-	3 (27.3)
• Electrocardiogram QT prolonged	-	-	-	3 (50.0)	-	3 (27.3)
• Diastolic blood pressure decreased	2 (40.0)	-	-	-	-	2 (18.2)
**Infections and infestations**						
**Viral infectious disorders**	**57 (39.3)**	**4 (36.4)**	**0 (0)**	**34 (55.7)**	**1 (100)**	**96 (43.6)**
• COVID-19	11 (19.3)	1 (25.0)	-	15 (44.1)	-	27 (28.1)
• Progressive multifocal leukoencephalopathy	14 (24.6)	-	-	5 (14.7)	-	19 (19.8)
• Herpes zoster	8 (14.0)	2 (50.0)	-	4 (11.8)	-	14 (14.6)
**Infections - pathogen unspecified**	**44 (30.3)**	**5 (45.5)**	**2 (100)**	**20 (32.8)**	**0 (0)**	**71 (32.3)**
• Urinary tract infection	9 (20.5)	3 (60.0)	1 (50.0)	9 (45.0)	-	22 (31.0)
• Infection	6 (13.6)	-	-	2 (10.0)	-	8 (11.3)
• Pneumonia	3 (6.8)	-	1 (50.0)	2 (10.0)	-	6 (8.5)
**Fungal infectious disorders**	**36 (24.8)**	**0 (0)**	**0 (0)**	**1 (1.6)**	**0 (0)**	**37 (16.8)**
• Cryptococcosis	10 (27.8)	-	-	-	-	10 (27.0)
• Histoplasmosis disseminated	9 (25.0)	-	-	-	-	9 (24.3)
• Meningitis cryptococcal	6 (16.7)	-	-	-	-	6 (16.2)

Data are expressed as N (%)

### RORs of lymphopenia

**Fig. 2:**
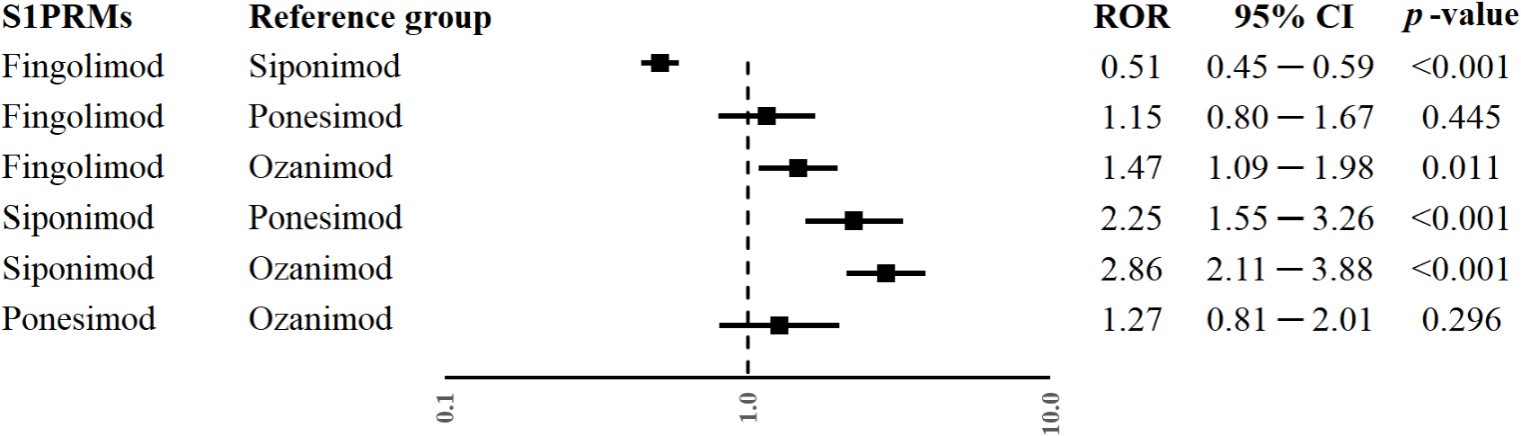
Odds ratio (ROR) of lymphopenia for each sphingosine 1-phosphate receptor modulator (S1PRMs) compared to each other. CI, confidence interval

Fingolimod was associated with a lower reporting frequency of lymphopenia compared to siponimod (ROR 0.51; 95% CI 0.45─0.59; p-value < 0.001). Moreover, siponimod and fingolimod were associated with a higher reporting frequency of lymphopenia compared to ozanimod (ROR 2.86; 95% CI 2.11─3.88; p-value < 0.001 and ROR 1.47; 95% CI 1.09─1.98; p-value = 0.011). Furthermore, a higher reporting probability of lymphopenia was also found when siponimod was compared to ponesimod (ROR 2.25; 95% CI 1.55─3.26; p-value = < 0.001). All RORs are shown in Fig. 2. The combination of fingolimod-siponimod was reported at least 3 times. No significant differences were observed for each S1PRMs compared to the combination fingolimod-siponimod (Supplementary Fig. 1).

### Post-hoc power calculations

Post-hoc power calculations were conducted to confirm the ability of the disproportionality analysis to detect significant differences between treatments. Statistical power was estimated for each comparison, with a conventional power threshold of 0.80 considered adequate. Calculations were performed using R (version 4.2.2, R Development Core Team). The results indicated high power for the comparisons between fingolimod and siponimod (1.00), siponimod and ponesimod (0.9904992), and siponimod and ozanimod (0.9999998). Conversely, lower power was observed for the comparisons between fingolimod and ponesimod (0.1037), and ponesimod and ozanimod (0.1523167). Moreover, a moderate power (0.6999) was found when fingolimod was compared to ozanimod.

## Discussion

In our database-related pharmacovigilance study, we investigated the reporting of lymphopenia in people with MS treated with S1PRMs (fingolimod, siponimod, ozanimod, and ponesimod) by analysing data from the EV database. To provide a descriptive analysis of this event, we have retrieved more than 5 thousand ICSRs relating to the period from January 1st, 2022, to December 31st, 2024, and filtered for the event of our interest (*N* = 949). Lymphopenia covered a relevant percentage (about 17%) of all ICSRs by the above-cited S1PRMs collected in the EV database. This result is in line with the fact that this event is related to the mechanism of action of S1PRMs. In registrational trials, the risk of lymphopenia associated with S1PRMs in people with MS has been well documented. In pivotal trials of fingolimod, such as FREEDOMS and TRANSFORMS, lymphopenia was a common adverse event. In FREEDOMS, grade 3 or 4 lymphopenia (absolute lymphocyte count < 500 cells/mm³) was reported in > 25% of patients, with nadir lymphocyte counts observed within the first month of treatment. Recovery of lymphocyte counts to normal levels after discontinuation took 4–8 weeks [25–27]. The EXPAND trial, which evaluated siponimod in secondary progressive MS (SPMS), showed that 21% of patients experienced grade 3 lymphopenia. Unlike fingolimod, siponimod has a shorter half-life, allowing for faster lymphocyte recovery (1–10 days) [28]. The SUNBEAM and RADIANCE trials showed a lower risk of severe lymphopenia with ozanimod compared to fingolimod. Grade 3 lymphopenia occurred in < 3% of patients, and lymphocyte counts typically returned to baseline within 48–72 h after discontinuation [29, 30]. In the OPTIMUM trial, lymphopenia was observed in less than 5% of patients after ponesimod treatment, and lymphocyte recovery occurs within 1–2 weeks after stopping treatment [31].

The higher percentage of lymphopenia cases was observed in adult people with MS. Only 17 cases were related to children or adolescents, all of which were associated with fingolimod, the only S1PRM currently approved in Europe for use in people aged 10 years and older [32]. Moreover, three lymphopenia cases were identified in newborns, attributable to foetal exposure during pregnancy. In our study, reports from women accounted for 73.2%. These findings are largely consistent with the typical age of onset of MS and the greater prevalence of this condition in women compared to men [4, 33, 34]. Generally, ADRs occur more frequently and are more severe in women than in men due to gender-related differences in pharmacokinetic, immunological, and hormonal factors. In fact, the female sex appears to be a risk factor for developing ADRs, with a rate 1.5 to 1.7 times higher than that of males [35–37]. Additionally, a German multicenter, single-arm, open-label study of patients with RRMS treated with fingolimod demonstrated an increased risk of fingolimod-induced lymphopenia in underweight women with low baseline lymphocyte counts [38].

Nearly all ICSRs were reported by healthcare professionals, although it is not possible to distinguish between medical and non-medical reporters within the category of healthcare professionals. Given the seriousness of lymphopenia (approximately 60.0% of all ICSRs), healthcare professionals could be more likely to report more severe clinical events than minor ones [39–41]. However, the greater involvement of healthcare professionals could be coincidental and/or linked to other factors.

Since their market introduction, S1PRMs have been associated with lymphopenia due to their impact on the normal trafficking and distribution of lymphocytes. These drugs function by binding to S1P receptors on lymphocytes, which leads to the internalization and subsequent degradation of these receptors. As a result, lymphocytes are unable to detect the S1P gradient necessary for their egress from the thymus, secondary lymphoid organs, and bone marrow into the bloodstream [42, 43]. This mechanism is crucial in autoimmune disorders such as MS, where limiting the movement of autoreactive lymphocytes into target tissues can help mitigate disease activity [44]. However, the sequestration effect typically results in a decrease in total peripheral lymphocyte count, with recovery occurring upon discontinuation of the S1PRMs as receptor expression returns to normal levels.

A retrospective cohort study has shown that fingolimod can affect the normal functioning of immune cells, leading to grade 4 lymphopenia as a side effect among Korean people with MS. Additionally, the temporal pattern of lymphocyte count changes observed in patients treated with fingolimod typically indicates that lymphopenia tends to occur early in the course of treatment. However, over time, lymphocyte counts often stabilize, although they may remain lower than baseline levels [20]. Another real-world study demonstrated that grade 4 lymphopenia can occur at 1 month after siponimod administration in people with secondary progressive MS and cannot be predicted [21]. A comparative study evaluated the benefit-risk profiles of ozanimod and fingolimod in people with relapsing MS. The findings indicated that patients treated with ozanimod had a lower risk of lymphocyte count reduction compared to those treated with fingolimod [45]. Moreover, an observational study has highlighted that people with MS who experience severe lymphopenia due to fingolimod treatment may show elevated absolute lymphocyte counts when switched to ozanimod [23]. Consistent with the literature, our disproportionality analyses showed a higher reporting frequency of lymphopenia with fingolimod and siponimod compared to ozanimod. In contrast, our findings highlighted a lower reporting frequency of lymphopenia when fingolimod was compared to siponimod. This result warrants further investigation. A recent review described that grade IV lymphopenia occurred in 1% of people with MS treated with siponimod, while it was observed in up to 18% of people with MS treated with fingolimod [46]. Fingolimod is a nonselective S1PRM, affecting multiple receptor subtypes (S1P1, S1P3, S1P4, and S1P5), whereas siponimod, ozanimod, and ponesimod are selective for the S1P1 and S1P5 subtypes [47]. This selectivity may result in a favourable side effect profile because it can reduce off-target effects associated with other S1P receptor subtypes [48]. Additionally, siponimod treatment is initiated with a 5-day titration pack, starting with 0.25 mg on days 1 and 2, followed by 0.5 mg on day 3, 0.75 mg on day 4, and 1.25 mg on day 5, before reaching the maintenance dose of 2 mg on day 6 [11]. This graduated dose titration protocol may contribute to a lower incidence of severe lymphopenia, as it allows for adaptive immune modulation and reduces abrupt changes in lymphocyte counts. However, the lower reporting frequency of lymphopenia for fingolimod compared to siponimod may be attributed to the fact that lymphopenia is a well-documented and expected effect of fingolimod, typically reported in more severe cases or when accompanied by other adverse outcomes. In contrast, the graduated dose titration protocol of siponimod may make lymphopenia a less anticipated occurrence, potentially leading to a higher reporting frequency. Based on the literature, siponimod, ozanimod, and ponesimod have been associated with a reduced impact on lymphocyte count, leading to a lower risk of higher-grade lymphopenia compared to fingolimod [49]. Moreover, the time it takes for lymphocytes to return to normal levels after stopping S1PRMs varies among the drugs. For fingolimod, it takes about 6 weeks, while for siponimod, it takes between 1 and 10 days. For ozanimod, it only takes 48–72 h, and for ponesimod, it takes 1–2 weeks. The newer S1PRMs have a shorter half-life than fingolimod, which helps lymphocytes recover more quickly after the medication is stopped [50–52]. However, a case report described a 55-year-old man who switched from fingolimod to siponimod and developed severe lymphopenia within a month [53]. The patient’s lymphocyte count dropped to 200/mmc after starting siponimod, despite a one-year interval from stopping fingolimod, which had maintained his lymphocyte count at a minimum of 530/mmc [53].

We also performed post-hoc power calculations in our pharmacovigilance study, acknowledging both their utility and inherent limitations. While post-hoc power analysis does not alter the initial results, it provides insight into the likelihood of detecting true differences, helping to contextualize our findings. Our power calculations were largely consistent with the p-values observed, further reinforcing the robustness of the significant comparisons. Notably, high statistical power was achieved for fingolimod versus siponimod, siponimod versus ponesimod, and siponimod versus ozanimod, supporting the reliability of these findings. However, lower power was observed in the comparisons between fingolimod and ponesimod, and between ponesimod and ozanimod, aligning with their non-significant p-values and indicating a higher likelihood of type II error (false negative). The comparison between fingolimod and ozanimod showed moderate power (0.699), suggesting a reasonable, though not definitive, ability to detect a true difference. While the p-value was significant, the suboptimal power indicates that the effect size estimate may be imprecise, warranting cautious interpretation.

Lymphopenia compromises the immune system’s ability. In our dataset, a significant proportion of other events reported in ICSRs belonged to the SOC “Infections and infestations”. The occurrence of opportunistic infections such as PML accounted for 19.8% of viral infections. PML is a severe condition associated with prolonged immunosuppression and has been reported in patients receiving DMTs, including natalizumab and fingolimod [54–56]. A key insight from our data is that the high frequency of common infections, such as upper respiratory tract infections and urinary tract infections, could potentially mask early signals of rarer but clinically significant opportunistic infections, such as PML. However, there does not appear to be a direct link between lymphopenia and infections. A systematic review and meta-analysis of 8,448 patients from 12 randomized controlled trials suggested an increased risk of infections with fingolimod but did not directly link this to lymphopenia severity [57]. Moreover, a recent pharmacovigilance study analyzing data from the FDA Adverse Event Reporting System (FAERS) indicated that infections were among the top reported adverse events associated with S1PRMs, emphasizing the importance of monitoring patients on these therapies, particularly those with lymphopenia [58]. The different nature of these infections underscores the need for monitoring strategies to mitigate the potentially serious consequences of compromised immune function in this patient population. Management of MS requires a personalized approach, considering factors such as patient demographics, disease severity, and comorbidities [59]. Regular monitoring of lymphocyte counts is essential during treatment with S1PRMs to detect and manage promptly lymphopenia. Treatment interruptions or modifications may be necessary to mitigate risks associated with severe lymphopenia. In addition to the occurrence of infections, our analysis also examined the relative absence of cancers. A real-world study showed an increased risk of reported skin cancer with S1PRMs, with the strongest association observed for basal cell carcinoma [60]. S1PRMs not only reduce circulating lymphocyte counts but also cause depletion of memory B cells and may affect other immune cell subsets that are crucial for tumor immune surveillance. MemoryB cells play an essential role in recognizing and eliminating early neoplastic cells, and their depletion may compromise the skin’s immune defense against tumor development [61]. Therefore, the increased incidence of basal cell carcinoma observed in some patients on S1PRMs might reflect these alternative immunological actions rather than being a direct consequence of lymphopenia. Further studies are warranted to delineate the precise pathways involved and to establish appropriate monitoring strategies for the early detection of skin cancers in this patient population.

### Strengths and limitations

Our pharmacovigilance study has several strengths and limitations. First, among its strengths, it plays a crucial role in improving our understanding of some aspects of the safety profile of S1PRMs in the real-world context. EudraVigilance is one of the largest pharmacovigilance databases, collecting drug safety data from different countries and populations. This enables comprehensive analysis of drug safety across diverse demographic groups and health conditions, providing insights that may not emerge in clinical trials alone. Additionally, pharmacovigilance facilitates the early detection of rare or unexpected adverse events that may occur post-marketing and enhances our ability to identify potential associations between medications and adverse events [62, 63]. Together with the other available evidence, this approach can support regulatory agencies in promptly assessing and responding to emerging safety concerns, thereby ensuring continued patient safety and informed medical decision-making. However, the disproportionality analysis applied in pharmacovigilance studies can only identify correlations, not causation. As a result, it cannot establish a definitive cause-and-effect relationship or replace clinical judgment for the individual patient. Therefore, it is important to interpret these findings appropriately without making claims on causal inference. Our study also has some limitations. Firstly, underreporting is a significant issue inherent in spontaneous reporting systems. Healthcare professionals, patients, and pharmaceutical companies often fail to report ADRs due to inadequate awareness of reporting requirements or uncertainty about the drug-event relationship. For instance, lymphopenia associated with fingolimod may be underreported, as it is an expected outcome of treatment and may only be documented when it is severe or linked to another adverse event. Conversely, lymphopenia with siponimod may be reported more frequently, as it is a less commonly observed effect and may be viewed as more noteworthy or surprising. Bias risk is also a concern. In particular, we are unable to determine the exact number of people with MS who received S1PRMs, but rather, we can only provide a general overview of cases associated with lymphopenia. Moreover, we focused only on people with MS, excluding cases that lacked a therapeutic indication or indicating a condition other than MS and its phenotypes. Additionally, events related to lymphopenia, such as infections or other immune system disorders, may not have been included. Therefore, we did not describe all possible exposure and outcome scenarios involving the four S1PRMs under consideration. Another important limitation is the variations in the quality and completeness of the EV data. In fact, the temporal relationship between exposure to S1PRMs and the onset of lymphopenia cannot be determined due to the lack of data regarding the time-to-onset among people with MS. Moreover, it is important to underline the presence of confounding factors, such as concomitant medications, underlying health conditions, and lifestyle factors, which can influence our findings. Notwithstanding these limitations, pharmacovigilance studies remain indispensable for ongoing post-market monitoring.

## Conclusions

This study provides an overview of spontaneous reports of lymphopenia in people with MS receiving S1PRMs (fingolimod, siponimod, ozanimod, and ponesimod). It highlights the importance of monitoring lymphocyte counts in people with MS treated with these drugs. Fingolimod and siponimod have shown a higher reporting frequency of lymphopenia. Conversely, the reporting of this event was less frequent for ozanimod and ponesimod, and this observation could suggest the possibility of investigating on what S1PRMs may be a safer option in terms of lymphopenia risk. Moreover, particular attention should be given to the early detection of opportunistic infections such as PML, which, although rare, can have severe consequences in immunosuppressed patients. The most important clinical implication of the disproportionality analysis applied to our data is to increase the awareness of the risk of lymphopenia related to S1P receptor modulators, thus supporting proactive monitoring and finally promoting safer prescribing. However, given the retrospective nature of our study, these results should be interpreted with caution. Further studies with mote rigorous study designs are needed to deepen these findings and to optimize treatment strategies for people with MS.

## Electronic supplementary material

Below is the link to the electronic supplementary material.

Supplementary Material 1

## Data Availability

The data that support the findings of this study are openly available in EudraVigilance (European database of suspected adverse drug reaction reports) at https://www.adrreports.eu/.

## Abbreviations

ADR: Adverse Drug Reaction
AEFI: Adverse Event Following Immunization
DMT: Disease–Modifying Therapy
EMA: European Medicines Agency
EV: EudraVigilance
FAERS: FDA Adverse Event Reporting System
FDA: Food and Drug Administration
HLGT: High–Level Group Term
HLT: High–Level Term
ICSR: Individual Case Safety Report
LFT: Liver Function Test
LLT: Lowest Level Term
MedDRA: Medical Dictionary for Regulatory Activities
MS: Multiple Sclerosis
MSIF: Multiple Sclerosis International Federation
PML: Progressive Multifocal Leukoencephalopathy
PT: Preferred Term
READUS: PV–REporting of A Disproportionality analysis for drUg Safety signal detection using ICSRs in PharmacoVigilance
ROR: Reporting Odds Ratio
S1PRM: Sphingosine 1–Phosphate Receptor Modulator
SOC: System Organ Class
SPMS: Secondary Progressive Multiple Sclerosis
RRMS: Relapsing–Remitting Multiple Sclerosis
VZV: Varicella Zoster virus
