# Supplementary material for: Lymphopenia associated with sphingosine 1-phosphate receptor modulators (S1PRMs) in multiple sclerosis: analysis of European pharmacovigilance data
Source: Pharmacol Rep. 2025 Apr 9;77(3):775–88. doi: 10.1007/s43440-025-00725-6 (PMC12066379; doi:10.1007/s43440-025-00725-6)

**Supplementary Table 1.** The REporting of A Disproportionality analysis for drUg Safety signal detection using individual case safety reports in PharmacoVigilance (READUS-PV) checklist.

| **Section and topic** | **Item #** | **Checklist item** | **Location where item is reported** |
| --- | --- | --- | --- |
| **Title** |  |  |  |
|  | *1a* | *If disproportionality analyses are a prominent component of the published study, the study should be identified as a “disproportionality analysis”. The type of data and name of the database(s) should be specified.* | No. We specified them in the section 2.5. Disproportionality Analysis. |
|  | *1b* | *Report the name of adverse event(s) and/or drug(s) under study, when applicable.* | Yes. |
| **Introduction** |  |  |  |
| Background | *2a* | *Describe the drug(s) and its utilization, the nature of the adverse event(s) under study and its frequency, and the existing knowledge on the drug-event combination.* | Yes. |
|  | *2b* | *Specify the rationale for performing the analysis, e.g., as part of routine pharmacovigilance, to investigate an overall safety profile, or to assess a pre-specified hypothesis.* | Yes. |
|  | *2c* | *Explain why ICSR databases and disproportionality analysis are suitable to fill the knowledge gap.* | Yes. |
| Objectives | *3* | *State specific objectives, identifying the adverse event(s), the drug(s), and the reference group, including any pre-specified hypothesis, if applicable.* | Yes. |
| **Methods** |  |  |  |
| Study design | *4a* | *Identify the study (i.e., “disproportionality analysis”) and the type of data used (e.g., “individual case safety reports”).* | Yes. We specified them in the sections 2.1. Study design, and 2.2. Data source |
|  | *4b* | *Provide an outline of the entire study design, including primary and sensitivity analyses performed, and other designs such as case-by-case analysis or literature review.* | Yes. We specified them in the section 2.4 Descriptive Analysis, and 2.5. Disproportionality Analysis. |
| Data description, access,  and pre-processing | *5a* | *Specify the name of the database(s), the database(s) custodian, and the coverage. Specify the type/number of drugs included within the database and the thesaurus, taxonomies, or ontologies used for coding drugs and events.* | Yes. We specified them in the section 2.2. Data source. |
|  | *5b* | *Specify the extraction dates and describe and justify all choices used for data pre-processing, including any data transformation or exclusion, if appropriate.* | Yes. We specified them in the section 2.3. ICSRs selection. |
| Variables definition | *6a* | *Describe the study population, including any restriction.* | Yes. We specified them in the sections 2.3. ICSRs selection and 2.4. Descriptive Analysis. |
|  | *6b* | *Describe the nature and the meaning of key variables assessed in the work.* | Yes. We specified them in the section 2.4. Descriptive Analysis. |
|  | *6c* | *Specify and justify any grouping of drugs or events. For drugs, specify and justify whether active ingredients/trade names/salts were considered and/or the selected role.* | Yes. We specified them in the sections 2.2. Data source and 2.3. ICSRs selection. |
|  | *6d* | *Describe any additional data source used, the type of data, and how they interact with ICSRs.* | No. We did not use any additional data source. |
| Statistical methods | *7a* | *Present any descriptive analysis performed, specifying variables investigated, statistical tests, and significance thresholds.* | Yes. We specified them in the section 2.4. Descriptive Analysis. |
|  | *7b* | *Describe the measure(s) selected for the disproportionality analysis including any threshold used to identify signals of disproportionate reporting. Explain the reason for this choice if applicable.* | Yes. We specified them in the section 2.5. Disproportionality Analysis. |
|  | *7c* | *Clearly describe any sensitivity analysis and any tool to control confounding, including any restriction, subgroup, stratification, adjustment, or interaction.* | No. |
|  | *7d* | *Specify the variables and methods used for the case-by-case analysis, including any algorithm or criteria used to assess causality, if performed.* | No. |
|  | *7e* | *Specify any statistical methods used for other data sources.* | No. |
| **Results** |  |  |  |
| Participants | *8a* | *Specify the number of individual case safety reports included at each stage, including reasons for exclusion.* | Yes. We specified them in the section 3. Results  and in Figure 1. |
|  | *8b* | *Provide key demographic and clinical characteristics of cases, if possible comparing cases with any appropriate reference group.* | Yes. We specified them in the section 3. Results. |
| Disproportionality analysis | *9* | *Present all results including confidence intervals. Present also results of sensitivity analyses, if performed.* | Yes. We specified them in the section 3.1 RORs of lymphopenia. |
| Case-by-case analysis | *10* | *Present the case-by-case analysis of key variables. Present the causality assessment, if applicable.* | No. |
| **Discussion** |  |  |  |
| Key results | *11* | *Discuss key results with reference to study objectives and contextualize them within the current literature and other consulted sources. Clearly discriminate between expected reactions and emerging safety signals.* | Yes. |
| External validity | *12a* | *Discuss the external validity of the results to the general population.* | Yes. |
|  | *12b* | *Discuss the potential relevance of results in clinical practice* | Yes. |
|  | *12c* | *Propose further study designs if applicable* | Yes. We specified them in the section 5. Conclusions. |
| Limitations | *13* | *Present general limitations, making clear that disproportionality analysis alone cannot prove causation or measure incidence, and specific limitations, including confounding and reporting bias and efforts to mitigate them.* | Yes. We specified them in the section 4.1. Strengths and limitations. |
| **Declarations** |  |  |  |
|  | *14a* | *Provide the source of funding/sponsorship and the role of the funders/sponsors for the present study and for any original study on which the present article is based.* | No. |
|  | *14b* | *Clearly identify potential commercial and intellectual conflicts of interest (e.g., link to any drug/event investigated, whether financial, legal action, or software used).* | Yes. |
|  | *14c* | *Declare any institutional approval needed or granted in the investigation.* | No. |
|  | *14d* | *Include a statement on data availability, code availability (including the version of the statistical software used), and protocol registration.* | Yes. |

**Supplementary Table 2.** Distribution of concomitant drugs classified by the second level of the Anatomical Therapeutic Chemical (ATC) classification system reported in the Individual Case Safety Reports (ICSRs) related to lymphopenia and Sphingosine 1-phosphate receptor modulators retrieved from the EudraVigilance spontaneous reporting system from 1st January 2022 to 31th December 2024.

| ATC Code | Name | Overall (N=630) |
| --- | --- | --- |
| A02 | Drugs for acid related disorders |  |
| A03 | Drugs for functional gastrointestinal disorders | 2 (0.3%) |
| A04 | Antiemetics and antinauseants | 5 (0.8%) |
| A05 | Bile and liver therapy | 3 (0.5%) |
| A06 | Drugs for constipation | 10 (1.6%) |
| A07 | Antidiarrheals | 2 (0.3%) |
| A10 | Drugs used in diabetes | 44 (7.0%) |
| A11 | Vitamins | 57 (9.0%) |
| A12 | Mineral supplements | 4 (0.6%) |
| B03 | Antianemic preparations | 17 (2.7%) |
| B05 | Blood substitutes and perfusion solutions | 3 (0.5%) |
| C01 | Cardiac therapy | 2 (0.3%) |
| C02 | Antihypertensives | 4 (0.6%) |
| C03 | Diuretics | 8 (1.3%) |
| C04 | Peripheral vasodilators | 1 (0.2%) |
| C05 | Vasoprotectives | 2 (0.3%) |
| C07 | Beta-blocking agents | 8 (1.3%) |
| C08 | Calcium channel blockers | 17 (2.7%) |
| C09 | Agents acting on the renin–angiotensin system | 17 (2.7%) |
| C10 | Lipid-modifying agents | 26 (4.1%) |
| D05 | Antipsoriatics | 2 (0.3%) |
| D07 | Corticosteroids, dermatological preparations | 7 (1.1%) |
| D08 | Antiseptics and disinfectants | 1 (0.2%) |
| D11 | Other dermatological preparations | 3 (0.5%) |
| G02 | Other gynecologicals | 2 (0.3%) |
| G03 | Sex hormones and modulators of the genital system | 4 (0.6%) |
| G04 | Urologicals | 23 (3.7%) |
| H01 | Pituitary and hypothalamic hormones and analogues | 2 (0.3%) |
| H02 | Corticosteroids for systemic use | 1 (0.2%) |
| H03 | Thyroid therapy | 19 (3.0%) |
| J01 | Antibacterials for systemic use | 3 (0.5%) |
| J02 | Antimycotics for systemic use | 1 (0.2%) |
| J04 | Antimycobacterials | 1 (0.2%) |
| J07 | Vaccines | 2 (0.3%) |
| L01 | Antineoplastic agents | 2 (0.3%) |
| L02 | Endocrine therapy | 3 (0.5%) |
| L03 | Immunostimulants | 11 (1.7%) |
| L04 | Immunosuppressants | 24 (3.8%) |
| M01 | Antiinflammatory and antirheumatic products, non-steroids | 20 (3.2%) |
| M03 | Muscle relaxants | 27 (4.3%) |
| M05 | Drugs for treatment of bone diseases | 3 (0.5%) |
| N01 | Anesthetics | 1 (0.2%) |
| N02 | Analgesics | 22 (3.5%) |
| N03 | Antiepileptics | 49 (7.8%) |
| N04 | Anti-parkinson drugs | 7 (1.1%) |
| N05 | Psycholeptics | 29 (4.6%) |
| N06 | Psychoanaleptics | 46 (7.3%) |
| N07 | Other nervous system drugs | 20 (3.2%) |
| P01 | Antiprotozoals | 5 (0.8%) |
| P03 | Ectoparasiticides, incl. scabicides, insecticides and repellents | 1 (0.2%) |
| R01 | Nasal preparations | 2 (0.3%) |
| R02 | Throat preparations | 1 (0.2%) |
| R03 | Drugs for obstructive airway diseases | 2 (0.3%) |
| R06 | Antihistamines for systemic use | 4 (0.6%) |
| S01 | Ophthalmologicals | 29 (4.6%) |
| V03 | All other therapeutic products | 2 (0.3%) |

**Supplementary Table 3.** Distribution of all adverse events reported in Individual Case Safety Reports (ICSRs) with SP1 receptor modulators and lymphopenia retrieved from the EudraVigilance spontaneous reporting system from 1st January 2022 to 31th December 2024.

|  | **Fingolimod (N=1639)** | **Ozanimod (N=104)** | **Ponesimod (N=62)** | **Siponimod (N=1009)** | **Fingolimod/Siponimod (N=42)** | **Siponimod/Ozanimod (N=1)** | **Overall (N=2857)** |
| --- | --- | --- | --- | --- | --- | --- | --- |
| **Preferred Term** |  |  |  |  |  |  |  |
| Abdominal distension | 1 (0.1) | - | - | 1 (0.1) | - | - | 2 (0.1) |
| Abdominal pain | 2 (0.1) | - | - | - | - | - | 2 (0.1) |
| Abnormal behaviour | 1 (0.1) | - | - | - | - | - | 1 (0.0) |
| Abnormal loss of weight | 1 (0.1) | - | - | - | - | - | 1 (0.0) |
| Acalculia | 1 (0.1) | - | - | - | - | - | 1 (0.0) |
| Accidental exposure to product by child | 1 (0.1) | - | - | - | - | - | 1 (0.0) |
| Acrochordon | 2 (0.1) | - | - | - | - | - | 2 (0.1) |
| Acute kidney injury | 1 (0.1) | - | - | 1 (0.1) | - | - | 2 (0.1) |
| Agitation | 1 (0.1) | - | - | - | - | - | 1 (0.0) |
| Agranulocytosis | 1 (0.1) | - | - | - | - | - | 1 (0.0) |
| Agraphia | 2 (0.1) | - | - | - | - | - | 2 (0.1) |
| Alanine aminotransferase increased | 12 (0.7) | - | 1 (1.6) | 5 (0.5) | - | - | 18 (0.6) |
| Alexia | 1 (0.1) | - | - | - | - | - | 1 (0.0) |
| Alopecia | 1 (0.1) | - | - | - | - | - | 1 (0.0) |
| Amnesia | 1 (0.1) | - | - | - | - | - | 1 (0.0) |
| Anaemia | 1 (0.1) | - | - | 1 (0.1) | - | - | 2 (0.1) |
| Anal incontinence | 1 (0.1) | - | - | - | - | - | 1 (0.0) |
| Anisocytosis | 1 (0.1) | - | - | - | - | - | 1 (0.0) |
| Anogenital warts | 1 (0.1) | - | - | - | - | - | 1 (0.0) |
| Anosmia | 1 (0.1) | - | - | - | - | - | 1 (0.0) |
| Anti-JC virus antibody index | 5 (0.3) | - | - | - | 1 (2.4) | - | 6 (0.2) |
| Anxiety | 2 (0.1) | - | - | - | - | - | 2 (0.1) |
| Aphasia | 9 (0.5) | - | - | 1 (0.1) | - | - | 10 (0.4) |
| Aphthous ulcer | 2 (0.1) | - | - | 1 (0.1) | - | - | 3 (0.1) |
| Apraxia | 1 (0.1) | - | - | - | - | - | 1 (0.0) |
| Arthralgia | 1 (0.1) | - | - | 1 (0.1) | - | - | 2 (0.1) |
| Aspartate aminotransferase increased | 7 (0.4) | 1 (1.0) | - | - | - | - | 8 (0.3) |
| Asthenia | 10 (0.6) | 1 (1.0) | 1 (1.6) | 7 (0.7) | - | - | 19 (0.7) |
| Asthma | 1 (0.1) | - | - | - | - | - | 1 (0.0) |
| Ataxia | 10 (0.6) | - | - | 1 (0.1) | 1 (2.4) | - | 12 (0.4) |
| Atrial fibrillation | 1 (0.1) | - | - | - | - | - | 1 (0.0) |
| Attention deficit hyperactivity disorder | 1 (0.1) | - | - | - | - | - | 1 (0.0) |
| Avoidant personality disorder | 1 (0.1) | - | - | - | - | - | 1 (0.0) |
| Back pain | 1 (0.1) | - | - | 3 (0.3) | - | - | 4 (0.1) |
| Balance disorder | 2 (0.1) | - | - | 3 (0.3) | - | - | 5 (0.2) |
| Basal cell carcinoma | 2 (0.1) | - | - | 1 (0.1) | - | - | 3 (0.1) |
| Basophil count decreased | 1 (0.1) | - | - | - | - | - | 1 (0.0) |
| Basophil count increased | 1 (0.1) | - | - | - | - | - | 1 (0.0) |
| Bell's palsy | 1 (0.1) | - | - | - | - | - | 1 (0.0) |
| Bladder cancer | 1 (0.1) | - | - | - | - | - | 1 (0.0) |
| Bladder disorder | 3 (0.2) | - | - | 3 (0.3) | - | - | 6 (0.2) |
| Blepharal papilloma | 1 (0.1) | - | - | - | - | - | 1 (0.0) |
| Blindness | 1 (0.1) | - | - | 1 (0.1) | - | - | 2 (0.1) |
| Blindness unilateral | 1 (0.1) | - | - | - | - | - | 1 (0.0) |
| Blood albumin decreased | 1 (0.1) | - | - | - | - | - | 1 (0.0) |
| Blood cholesterol increased | 1 (0.1) | - | - | - | - | - | 1 (0.0) |
| Blood cholinesterase increased | 1 (0.1) | - | - | - | - | - | 1 (0.0) |
| Blood disorder | 1 (0.1) | - | 1 (1.6) | 1 (0.1) | - | - | 3 (0.1) |
| Blood immunoglobulin A decreased | 1 (0.1) | - | - | - | - | - | 1 (0.0) |
| Blood immunoglobulin M decreased | 1 (0.1) | - | - | - | - | - | 1 (0.0) |
| Blood magnesium increased | 1 (0.1) | - | - | - | - | - | 1 (0.0) |
| Blood pressure diastolic decreased | 2 (0.1) | - | - | - | - | - | 2 (0.1) |
| Blood pressure fluctuation | 1 (0.1) | - | - | - | - | - | 1 (0.0) |
| Blood pressure increased | 1 (0.1) | - | - | 2 (0.2) | - | - | 3 (0.1) |
| Blood test abnormal | 3 (0.2) | - | - | - | - | - | 3 (0.1) |
| Body temperature increased | 1 (0.1) | - | - | - | - | - | 1 (0.0) |
| Bone lesion | 1 (0.1) | - | - | - | - | - | 1 (0.0) |
| Bradycardia | 1 (0.1) | 1 (1.0) | - | 3 (0.3) | 2 (4.8) | - | 7 (0.2) |
| Brain herniation | 2 (0.1) | - | - | - | - | - | 2 (0.1) |
| Brain stem syndrome | 1 (0.1) | - | - | - | - | - | 1 (0.0) |
| Breast cancer | 1 (0.1) | - | - | - | - | - | 1 (0.0) |
| Breast cancer stage I | 1 (0.1) | - | - | - | - | - | 1 (0.0) |
| Brudzinski's sign | 1 (0.1) | - | - | - | - | - | 1 (0.0) |
| Burning sensation | 2 (0.1) | - | - | - | - | - | 2 (0.1) |
| C-reactive protein increased | 1 (0.1) | - | - | - | - | - | 1 (0.0) |
| Carbohydrate antigen 125 increased | 1 (0.1) | - | - | - | - | - | 1 (0.0) |
| Cardiac disorder | 4 (0.2) | - | - | - | - | - | 4 (0.1) |
| Cardiac failure | 2 (0.1) | - | - | - | - | - | 2 (0.1) |
| Cardiomegaly | 1 (0.1) | - | - | - | - | - | 1 (0.0) |
| Cartilage injury | 1 (0.1) | - | - | - | - | - | 1 (0.0) |
| Cataract subcapsular | 1 (0.1) | - | - | - | - | - | 1 (0.0) |
| Cellulitis | 5 (0.3) | - | - | - | - | - | 5 (0.2) |
| Central nervous system infection | 1 (0.1) | - | - | - | - | - | 1 (0.0) |
| Central nervous system lesion | 9 (0.5) | - | - | 2 (0.2) | 1 (2.4) | - | 12 (0.4) |
| Cerebellar ischaemia | 1 (0.1) | - | - | - | - | - | 1 (0.0) |
| Cerebral atrophy | 2 (0.1) | - | - | - | 1 (2.4) | - | 3 (0.1) |
| Cerebral disorder | 1 (0.1) | - | - | - | - | - | 1 (0.0) |
| Cerebral haemorrhage | 2 (0.1) | - | - | - | - | - | 2 (0.1) |
| Cerebrovascular accident | 1 (0.1) | - | - | - | - | - | 1 (0.0) |
| Cervical dysplasia | 2 (0.1) | - | - | - | - | - | 2 (0.1) |
| Cervix carcinoma stage II | 1 (0.1) | - | - | - | - | - | 1 (0.0) |
| Chest discomfort | 1 (0.1) | - | - | - | - | - | 1 (0.0) |
| Chest pain | 2 (0.1) | - | - | - | - | - | 2 (0.1) |
| Chest wall mass | 1 (0.1) | - | - | - | - | - | 1 (0.0) |
| Chills | 1 (0.1) | - | - | 1 (0.1) | - | - | 2 (0.1) |
| Chronic respiratory failure | 1 (0.1) | - | - | - | - | - | 1 (0.0) |
| Clumsiness | 3 (0.2) | - | - | - | - | - | 3 (0.1) |
| Cognitive disorder | 8 (0.5) | - | - | 2 (0.2) | 1 (2.4) | - | 11 (0.4) |
| Colitis ulcerative | 1 (0.1) | - | - | - | - | - | 1 (0.0) |
| Combined immunodeficiency | 1 (0.1) | - | - | - | - | - | 1 (0.0) |
| Communication disorder | 1 (0.1) | - | - | - | - | - | 1 (0.0) |
| Concomitant disease progression | 1 (0.1) | - | - | 1 (0.1) | - | - | 2 (0.1) |
| Condition aggravated | 8 (0.5) | - | - | 2 (0.2) | - | - | 10 (0.4) |
| Confusional state | 2 (0.1) | 1 (1.0) | - | 1 (0.1) | - | - | 4 (0.1) |
| Constipation | 1 (0.1) | - | - | 3 (0.3) | - | - | 4 (0.1) |
| Contusion | 1 (0.1) | - | - | - | - | - | 1 (0.0) |
| Coordination abnormal | 2 (0.1) | - | - | - | - | - | 2 (0.1) |
| Coronavirus infection | 2 (0.1) | - | - | 2 (0.2) | - | - | 4 (0.1) |
| Cough | 3 (0.2) | - | - | 1 (0.1) | - | - | 4 (0.1) |
| COVID-19 | 11 (0.7) | 1 (1.0) | - | 15 (1.5) | - | - | 27 (0.9) |
| Cranial nerve disorder | 1 (0.1) | - | - | - | - | - | 1 (0.0) |
| Cranial nerve injury | 1 (0.1) | - | - | - | - | - | 1 (0.0) |
| Cryptococcal cutaneous infection | 3 (0.2) | - | - | - | - | - | 3 (0.1) |
| Cryptococcal meningoencephalitis | 1 (0.1) | - | - | - | - | - | 1 (0.0) |
| Cryptococcosis | 10 (0.6) | - | - | - | - | - | 10 (0.4) |
| CSF protein increased | 1 (0.1) | - | - | - | - | - | 1 (0.0) |
| Cyst | 2 (0.1) | - | - | - | - | - | 2 (0.1) |
| Cystoid macular oedema | 1 (0.1) | - | - | - | - | - | 1 (0.0) |
| Decreased gait velocity | 2 (0.1) | - | - | - | - | - | 2 (0.1) |
| Decreased immune responsiveness | 4 (0.2) | - | - | - | - | - | 4 (0.1) |
| Decreased vibratory sense | 1 (0.1) | - | - | 1 (0.1) | - | - | 2 (0.1) |
| Defaecation disorder | 1 (0.1) | - | - | - | - | - | 1 (0.0) |
| Delirium | 1 (0.1) | - | - | 1 (0.1) | - | - | 2 (0.1) |
| Delivery | 1 (0.1) | - | - | - | - | - | 1 (0.0) |
| Dementia | 1 (0.1) | - | - | - | - | - | 1 (0.0) |
| Demyelination | 4 (0.2) | - | - | - | - | - | 4 (0.1) |
| Depression | 6 (0.4) | 1 (1.0) | - | - | - | - | 7 (0.2) |
| Dermatitis | 1 (0.1) | - | - | - | - | - | 1 (0.0) |
| Diabetes mellitus | 1 (0.1) | - | - | - | - | - | 1 (0.0) |
| Diabetes mellitus inadequate control | 1 (0.1) | - | - | - | - | - | 1 (0.0) |
| Diabetic retinal oedema | 1 (0.1) | - | - | - | - | - | 1 (0.0) |
| Diabetic retinopathy | 1 (0.1) | - | - | - | - | - | 1 (0.0) |
| Diarrhoea | 2 (0.1) | - | - | 2 (0.2) | - | - | 4 (0.1) |
| Diffuse alopecia | 1 (0.1) | - | - | - | - | - | 1 (0.0) |
| Diffuse large B-cell lymphoma | 3 (0.2) | - | - | - | - | - | 3 (0.1) |
| Diplopia | 5 (0.3) | - | - | - | - | - | 5 (0.2) |
| Discouragement | 1 (0.1) | - | - | - | - | - | 1 (0.0) |
| Disease progression | 4 (0.2) | - | - | - | 1 (2.4) | - | 5 (0.2) |
| Disease recurrence | 4 (0.2) | - | - | - | 1 (2.4) | - | 5 (0.2) |
| Disorientation | 2 (0.1) | - | - | - | - | - | 2 (0.1) |
| Disseminated cryptococcosis | 1 (0.1) | - | - | - | - | - | 1 (0.0) |
| Disturbance in attention | 1 (0.1) | - | - | 1 (0.1) | - | - | 2 (0.1) |
| Dizziness | 5 (0.3) | - | - | 5 (0.5) | - | - | 10 (0.4) |
| Dizziness postural | 1 (0.1) | - | - | - | - | - | 1 (0.0) |
| Drug-induced liver injury | 1 (0.1) | - | - | - | - | - | 1 (0.0) |
| Drug ineffective | 13 (0.8) | 1 (1.0) | - | 4 (0.4) | - | - | 18 (0.6) |
| Drug interaction | 1 (0.1) | - | - | 1 (0.1) | - | - | 2 (0.1) |
| Drug withdrawal syndrome | 1 (0.1) | - | - | - | - | - | 1 (0.0) |
| Dysaesthesia | 1 (0.1) | - | - | - | - | - | 1 (0.0) |
| Dysarthria | 8 (0.5) | - | - | 6 (0.6) | - | - | 14 (0.5) |
| Dyslalia | 4 (0.2) | - | - | - | - | - | 4 (0.1) |
| Dyslipidaemia | 1 (0.1) | - | - | - | - | - | 1 (0.0) |
| Dysmetria | 1 (0.1) | - | - | 7 (0.7) | - | - | 8 (0.3) |
| Dyspepsia | 1 (0.1) | - | - | - | - | - | 1 (0.0) |
| Dysphagia | 10 (0.6) | - | - | 4 (0.4) | - | - | 14 (0.5) |
| Dyspnoea | 2 (0.1) | - | 1 (1.6) | 2 (0.2) | - | - | 5 (0.2) |
| Dysuria | 2 (0.1) | - | - | 2 (0.2) | - | - | 4 (0.1) |
| Eczema | 1 (0.1) | - | - | - | - | - | 1 (0.0) |
| Encephalitis | 2 (0.1) | - | - | - | - | - | 2 (0.1) |
| Endometrial adenocarcinoma | 1 (0.1) | - | - | - | - | - | 1 (0.0) |
| Enterococcal infection | 1 (0.1) | - | - | - | - | - | 1 (0.0) |
| Eosinophil count decreased | 1 (0.1) | - | - | 1 (0.1) | - | - | 2 (0.1) |
| Epilepsy | 1 (0.1) | - | - | 3 (0.3) | 1 (2.4) | - | 5 (0.2) |
| Episcleritis | 1 (0.1) | - | - | - | - | - | 1 (0.0) |
| Epstein-Barr virus antigen positive | 1 (0.1) | - | - | - | - | - | 1 (0.0) |
| Epstein-Barr virus infection | 1 (0.1) | - | - | - | - | - | 1 (0.0) |
| Escherichia infection | 1 (0.1) | - | - | 1 (0.1) | - | - | 2 (0.1) |
| Escherichia urinary tract infection | 1 (0.1) | - | - | - | - | - | 1 (0.0) |
| Expanded disability status scale score increased | 2 (0.1) | - | - | 6 (0.6) | 1 (2.4) | - | 9 (0.3) |
| Extensor plantar response | 1 (0.1) | - | - | - | - | - | 1 (0.0) |
| Extradural haematoma | 1 (0.1) | - | - | - | - | - | 1 (0.0) |
| Eye haemorrhage | 1 (0.1) | - | - | - | - | - | 1 (0.0) |
| Eye pain | 3 (0.2) | - | - | 1 (0.1) | - | - | 4 (0.1) |
| Facet joint syndrome | 1 (0.1) | - | - | - | - | - | 1 (0.0) |
| Facial paralysis | 6 (0.4) | - | - | - | - | - | 6 (0.2) |
| Fall | 9 (0.5) | - | - | 4 (0.4) | - | - | 13 (0.5) |
| Fatigue | 19 (1.2) | 1 (1.0) | - | 13 (1.3) | - | - | 33 (1.2) |
| Fear of injection | 1 (0.1) | - | - | - | - | - | 1 (0.0) |
| Feeling abnormal | 4 (0.2) | - | - | 2 (0.2) | - | - | 6 (0.2) |
| Fine motor skill dysfunction | 1 (0.1) | - | - | - | - | - | 1 (0.0) |
| Foetal exposure during pregnancy | 3 (0.2) | - | - | - | - | - | 3 (0.1) |
| Follicular lymphoma | 1 (0.1) | - | - | - | - | - | 1 (0.0) |
| Foot deformity | 1 (0.1) | - | - | - | - | - | 1 (0.0) |
| Frontotemporal dementia | 1 (0.1) | - | - | - | - | - | 1 (0.0) |
| Full blood count abnormal | 2 (0.1) | - | - | - | - | - | 2 (0.1) |
| Gait disturbance | 13 (0.8) | - | - | 13 (1.3) | 3 (7.1) | - | 29 (1.0) |
| Gait inability | 1 (0.1) | - | - | - | - | - | 1 (0.0) |
| Gamma-glutamyltransferase increased | 12 (0.7) | 1 (1.0) | 1 (1.6) | 10 (1.0) | - | - | 24 (0.8) |
| Gastroenteritis | 1 (0.1) | - | - | - | - | - | 1 (0.0) |
| Gastrointestinal disorder | 2 (0.1) | - | - | - | - | - | 2 (0.1) |
| Gastrointestinal haemorrhage | 5 (0.3) | - | - | - | - | - | 5 (0.2) |
| Gastrointestinal infection | 5 (0.3) | - | - | - | - | - | 5 (0.2) |
| General physical health deterioration | 1 (0.1) | - | - | 1 (0.1) | - | - | 2 (0.1) |
| Generalised anxiety disorder | 1 (0.1) | - | - | - | - | - | 1 (0.0) |
| Glioblastoma | 1 (0.1) | - | - | - | - | - | 1 (0.0) |
| Granulocytes abnormal | 1 (0.1) | - | - | - | - | - | 1 (0.0) |
| Haemangioma | 1 (0.1) | - | - | - | - | - | 1 (0.0) |
| Haematocrit decreased | 3 (0.2) | - | - | 1 (0.1) | - | - | 4 (0.1) |
| Haematotoxicity | 1 (0.1) | - | - | - | - | - | 1 (0.0) |
| Haematuria | 5 (0.3) | - | - | 7 (0.7) | - | - | 12 (0.4) |
| Haemoglobin decreased | 1 (0.1) | - | - | 1 (0.1) | - | - | 2 (0.1) |
| Haemorrhagic stroke | 1 (0.1) | - | - | - | - | - | 1 (0.0) |
| Hallucination | 1 (0.1) | - | - | - | - | - | 1 (0.0) |
| Halo vision | 1 (0.1) | - | - | - | - | - | 1 (0.0) |
| Head discomfort | 1 (0.1) | - | - | - | - | - | 1 (0.0) |
| Headache | 11 (0.7) | - | 1 (1.6) | 7 (0.7) | - | - | 19 (0.7) |
| Heart rate abnormal | 1 (0.1) | - | - | - | - | - | 1 (0.0) |
| Heart rate increased | 1 (0.1) | - | - | - | - | - | 1 (0.0) |
| Hemianopia | 1 (0.1) | - | - | - | - | - | 1 (0.0) |
| Hemihypoaesthesia | 2 (0.1) | - | - | - | - | - | 2 (0.1) |
| Hemiparesis | 5 (0.3) | - | - | 4 (0.4) | 1 (2.4) | - | 10 (0.4) |
| Hemiplegia | 2 (0.1) | - | - | - | - | - | 2 (0.1) |
| Hepatic cyst | 1 (0.1) | - | - | - | - | - | 1 (0.0) |
| Hepatic enzyme abnormal | 1 (0.1) | - | - | - | - | - | 1 (0.0) |
| Hepatic enzyme increased | 9 (0.5) | 1 (1.0) | 2 (3.2) | 5 (0.5) | - | - | 17 (0.6) |
| Hepatic function abnormal | 2 (0.1) | - | - | 2 (0.2) | 1 (2.4) | - | 5 (0.2) |
| Hepatic lesion | 1 (0.1) | - | - | - | - | - | 1 (0.0) |
| Hepatic steatosis | 1 (0.1) | - | - | 1 (0.1) | - | - | 2 (0.1) |
| Hepatobiliary disease | 1 (0.1) | - | - | - | - | - | 1 (0.0) |
| Hepatocellular injury | 1 (0.1) | - | - | - | - | - | 1 (0.0) |
| Hepatosplenomegaly | 1 (0.1) | - | - | - | - | - | 1 (0.0) |
| Herpes dermatitis | 1 (0.1) | - | - | - | - | - | 1 (0.0) |
| Herpes virus infection | 3 (0.2) | - | - | - | - | - | 3 (0.1) |
| Herpes zoster | 8 (0.5) | 2 (1.9) | - | 4 (0.4) | - | - | 14 (0.5) |
| Herpes zoster meningitis | 1 (0.1) | - | - | - | - | - | 1 (0.0) |
| Herpes zoster meningoencephalitis | 1 (0.1) | - | - | - | - | - | 1 (0.0) |
| Herpes zoster oticus | 1 (0.1) | - | - | - | - | - | 1 (0.0) |
| Herpetic radiculopathy | 1 (0.1) | - | - | - | - | - | 1 (0.0) |
| Hiccups | 1 (0.1) | - | - | - | - | - | 1 (0.0) |
| Histoplasmosis disseminated | 9 (0.5) | - | - | - | - | - | 9 (0.3) |
| Hydrocephalus | 1 (0.1) | 1 (1.0) | - | - | - | - | 2 (0.1) |
| Hyperbilirubinaemia | 4 (0.2) | - | - | - | - | - | 4 (0.1) |
| Hypercalcaemia | 1 (0.1) | - | - | - | - | - | 1 (0.0) |
| Hypercholesterolaemia | 1 (0.1) | - | - | - | - | - | 1 (0.0) |
| Hyperhidrosis | 1 (0.1) | - | 1 (1.6) | - | - | - | 2 (0.1) |
| Hyperpyrexia | 1 (0.1) | - | - | - | - | - | 1 (0.0) |
| Hyperreflexia | 3 (0.2) | - | - | - | - | - | 3 (0.1) |
| Hypersensitivity | 1 (0.1) | - | - | 1 (0.1) | - | - | 2 (0.1) |
| Hypertension | 7 (0.4) | - | - | 1 (0.1) | - | - | 8 (0.3) |
| Hypoaesthesia | 19 (1.2) | - | - | 1 (0.1) | 1 (2.4) | - | 21 (0.7) |
| Hypoaesthesia oral | 1 (0.1) | - | - | - | - | - | 1 (0.0) |
| Hyporesponsive to stimuli | 1 (0.1) | - | - | - | - | - | 1 (0.0) |
| Hypothermia | 1 (0.1) | - | - | - | - | - | 1 (0.0) |
| Hysterectomy | 1 (0.1) | - | - | - | - | - | 1 (0.0) |
| Immune reconstitution inflammatory syndrome | 8 (0.5) | 1 (1.0) | - | - | - | - | 9 (0.3) |
| Immune system disorder | 3 (0.2) | - | - | - | - | - | 3 (0.1) |
| Immunodeficiency | 1 (0.1) | 2 (1.9) | - | - | - | - | 3 (0.1) |
| Immunosuppression | 1 (0.1) | - | - | - | - | - | 1 (0.0) |
| Impaired healing | 3 (0.2) | - | - | 1 (0.1) | - | - | 4 (0.1) |
| Inappropriate schedule of product administration | 9 (0.5) | - | - | 4 (0.4) | - | - | 13 (0.5) |
| Incontinence | 1 (0.1) | - | - | 3 (0.3) | - | - | 4 (0.1) |
| Incorrect dose administered | 1 (0.1) | - | - | 2 (0.2) | - | - | 3 (0.1) |
| Increased appetite | 1 (0.1) | - | - | - | - | - | 1 (0.0) |
| Infection | 6 (0.4) | - | - | 2 (0.2) | - | - | 8 (0.3) |
| Inflammation | 2 (0.1) | - | - | 1 (0.1) | - | - | 3 (0.1) |
| Influenza | 1 (0.1) | - | - | 2 (0.2) | - | - | 3 (0.1) |
| Injury | 1 (0.1) | - | - | - | - | - | 1 (0.0) |
| Intentional underdose | 1 (0.1) | - | - | - | - | - | 1 (0.0) |
| Intermenstrual bleeding | 1 (0.1) | - | - | - | - | - | 1 (0.0) |
| Intermittent claudication | 1 (0.1) | - | - | - | - | - | 1 (0.0) |
| Intervertebral disc degeneration | 1 (0.1) | - | - | - | - | - | 1 (0.0) |
| Intervertebral disc disorder | 1 (0.1) | - | - | - | - | - | 1 (0.0) |
| Intervertebral disc protrusion | 2 (0.1) | - | - | - | - | - | 2 (0.1) |
| Invasive ductal breast carcinoma | 1 (0.1) | - | - | - | - | - | 1 (0.0) |
| Iridocyclitis | 1 (0.1) | - | - | - | - | - | 1 (0.0) |
| Iris adhesions | 1 (0.1) | - | - | - | - | - | 1 (0.0) |
| Ischaemic stroke | 1 (0.1) | - | - | - | - | - | 1 (0.0) |
| JC polyomavirus test positive | 11 (0.7) | - | - | - | - | - | 11 (0.4) |
| JC virus CSF test positive | 1 (0.1) | - | - | - | - | - | 1 (0.0) |
| JC virus infection | 1 (0.1) | - | - | 3 (0.3) | - | - | 4 (0.1) |
| Kernig's sign | 1 (0.1) | - | - | - | - | - | 1 (0.0) |
| Leukaemia | 1 (0.1) | - | - | - | - | - | 1 (0.0) |
| Leukopenia | 28 (1.7) | 6 (5.8) | 1 (1.6) | 15 (1.5) | - | - | 50 (1.8) |
| Limb discomfort | 3 (0.2) | - | - | 3 (0.3) | - | - | 6 (0.2) |
| Liver disorder | 1 (0.1) | - | - | 1 (0.1) | - | - | 2 (0.1) |
| Liver function test abnormal | 1 (0.1) | - | - | 2 (0.2) | - | - | 3 (0.1) |
| Liver function test increased | 5 (0.3) | - | - | 9 (0.9) | - | - | 14 (0.5) |
| Liver injury | 1 (0.1) | - | - | - | - | - | 1 (0.0) |
| Lordosis | 1 (0.1) | - | - | - | - | - | 1 (0.0) |
| Lumbar vertebral fracture | 1 (0.1) | - | - | - | - | - | 1 (0.0) |
| Lymphadenopathy | 4 (0.2) | - | - | - | - | - | 4 (0.1) |
| Lymphatic disorder | 4 (0.2) | - | 1 (1.6) | 1 (0.1) | - | - | 6 (0.2) |
| Lymphocyte count abnormal | 2 (0.1) | - | - | - | - | - | 2 (0.1) |
| Lymphocyte count decreased | 210 (12.8) | 11 (10.6) | 14 (22.6) | 185 (18.3) | 5 (11.9) | - | 425 (14.9) |
| Lymphocyte count increased | 1 (0.1) | - | - | 1 (0.1) | - | - | 2 (0.1) |
| Lymphopenia | 277 (16.9) | 39 (37.5) | 18 (29.0) | 184 (18.2) | 5 (11.9) | 1 (100) | 524 (18.3) |
| Macular oedema | 2 (0.1) | - | - | 2 (0.2) | - | - | 4 (0.1) |
| Maculopathy | 1 (0.1) | - | - | - | - | - | 1 (0.0) |
| Magnetic resonance imaging abnormal | 1 (0.1) | - | - | - | - | - | 1 (0.0) |
| Magnetic resonance imaging head abnormal | 1 (0.1) | - | - | - | - | - | 1 (0.0) |
| Malaise | 4 (0.2) | - | - | 1 (0.1) | - | - | 5 (0.2) |
| Malignant hypertension | 1 (0.1) | - | - | - | - | - | 1 (0.0) |
| Mass | 1 (0.1) | - | - | - | - | - | 1 (0.0) |
| Maternal exposure during pregnancy | 1 (0.1) | - | - | - | - | - | 1 (0.0) |
| Mean cell haemoglobin concentration decreased | 1 (0.1) | - | - | - | - | - | 1 (0.0) |
| Mean cell haemoglobin decreased | 1 (0.1) | - | - | - | - | - | 1 (0.0) |
| Mean platelet volume decreased | 2 (0.1) | - | - | - | - | - | 2 (0.1) |
| Melanocytic naevus | 2 (0.1) | - | - | - | - | - | 2 (0.1) |
| Memory impairment | 5 (0.3) | 1 (1.0) | - | 3 (0.3) | - | - | 9 (0.3) |
| Meningitis aseptic | 1 (0.1) | - | - | - | - | - | 1 (0.0) |
| Meningitis cryptococcal | 6 (0.4) | - | - | - | - | - | 6 (0.2) |
| Meningoradiculitis | 1 (0.1) | - | - | - | - | - | 1 (0.0) |
| Mental disorder | 1 (0.1) | - | - | 1 (0.1) | - | - | 2 (0.1) |
| Metabolic dysfunction-associated liver disease | 2 (0.1) | - | - | - | - | - | 2 (0.1) |
| Micturition urgency | 1 (0.1) | - | - | - | - | - | 1 (0.0) |
| Migraine | 2 (0.1) | - | - | 4 (0.4) | - | - | 6 (0.2) |
| Molluscum contagiosum | 3 (0.2) | - | - | - | - | - | 3 (0.1) |
| Monkeypox | 4 (0.2) | - | - | - | - | - | 4 (0.1) |
| Monocyte count decreased | 3 (0.2) | - | - | 1 (0.1) | - | - | 4 (0.1) |
| Monoplegia | 3 (0.2) | - | - | - | - | - | 3 (0.1) |
| Motor dysfunction | 3 (0.2) | - | - | - | - | - | 3 (0.1) |
| Movement disorder | 2 (0.1) | - | - | 3 (0.3) | - | - | 5 (0.2) |
| Multiple lentigines syndrome | 1 (0.1) | - | - | - | - | - | 1 (0.0) |
| Multiple sclerosis relapse | 71 (4.3) | - | 1 (1.6) | 24 (2.4) | 3 (7.1) | - | 99 (3.5) |
| Muscle spasms | 1 (0.1) | - | - | - | - | - | 1 (0.0) |
| Muscle spasticity | 4 (0.2) | - | - | 7 (0.7) | - | - | 11 (0.4) |
| Muscle strength abnormal | 1 (0.1) | - | - | - | - | - | 1 (0.0) |
| Muscular weakness | 8 (0.5) | - | - | 11 (1.1) | 1 (2.4) | - | 20 (0.7) |
| Myalgia | 1 (0.1) | - | 1 (1.6) | 1 (0.1) | - | - | 3 (0.1) |
| Myocardial infarction | 3 (0.2) | - | - | - | - | - | 3 (0.1) |
| Nasopharyngitis | 2 (0.1) | - | - | 1 (0.1) | - | - | 3 (0.1) |
| Nausea | 6 (0.4) | - | - | 2 (0.2) | - | - | 8 (0.3) |
| Neck pain | 1 (0.1) | - | - | 1 (0.1) | - | - | 2 (0.1) |
| Negative thoughts | 1 (0.1) | - | - | - | - | - | 1 (0.0) |
| Nervous system disorder | 1 (0.1) | - | - | 1 (0.1) | - | - | 2 (0.1) |
| Neurocryptococcosis | 1 (0.1) | - | - | - | - | - | 1 (0.0) |
| Neurologic neglect syndrome | 1 (0.1) | - | - | - | - | - | 1 (0.0) |
| Neurological symptom | 1 (0.1) | - | - | - | - | - | 1 (0.0) |
| Neutropenia | 10 (0.6) | 1 (1.0) | 1 (1.6) | 3 (0.3) | - | - | 15 (0.5) |
| Neutrophil count decreased | 2 (0.1) | 1 (1.0) | - | 4 (0.4) | - | - | 7 (0.2) |
| Neutrophil count increased | 3 (0.2) | - | - | 3 (0.3) | - | - | 6 (0.2) |
| Neutrophil toxic granulation present | 1 (0.1) | - | - | - | - | - | 1 (0.0) |
| Nocturia | 1 (0.1) | - | - | - | - | - | 1 (0.0) |
| Nodule | 1 (0.1) | - | - | - | - | - | 1 (0.0) |
| Normal newborn | 1 (0.1) | - | - | - | - | - | 1 (0.0) |
| Nuchal rigidity | 1 (0.1) | - | - | - | - | - | 1 (0.0) |
| Nystagmus | 3 (0.2) | - | - | - | - | - | 3 (0.1) |
| Obesity | 2 (0.1) | - | - | - | - | - | 2 (0.1) |
| Oedema peripheral | 1 (0.1) | 2 (1.9) | - | 3 (0.3) | - | - | 6 (0.2) |
| Off label use | 7 (0.4) | 1 (1.0) | - | 3 (0.3) | 1 (2.4) | - | 12 (0.4) |
| Ophthalmoplegia | 3 (0.2) | - | - | - | - | - | 3 (0.1) |
| Optic nerve disorder | 2 (0.1) | - | - | - | - | - | 2 (0.1) |
| Optic neuritis | 3 (0.2) | - | - | 1 (0.1) | - | - | 4 (0.1) |
| Oral herpes | 1 (0.1) | - | - | - | - | - | 1 (0.0) |
| Oropharyngeal discomfort | 1 (0.1) | - | - | - | - | - | 1 (0.0) |
| Osteomyelitis | 1 (0.1) | - | - | - | - | - | 1 (0.0) |
| Osteoporosis | 1 (0.1) | - | - | - | - | - | 1 (0.0) |
| Overweight | 1 (0.1) | - | - | - | - | - | 1 (0.0) |
| Pain | 3 (0.2) | - | - | 1 (0.1) | - | - | 4 (0.1) |
| Palpitations | 1 (0.1) | - | 1 (1.6) | - | - | - | 2 (0.1) |
| Papilloedema | 1 (0.1) | - | - | - | - | - | 1 (0.0) |
| Paraesthesia | 13 (0.8) | - | - | 1 (0.1) | - | - | 14 (0.5) |
| Paraesthesia oral | 1 (0.1) | - | - | - | - | - | 1 (0.0) |
| Paralysis recurrent laryngeal nerve | 1 (0.1) | - | - | - | - | - | 1 (0.0) |
| Paraparesis | 3 (0.2) | - | - | 1 (0.1) | - | - | 4 (0.1) |
| Paresis | 2 (0.1) | - | - | 2 (0.2) | - | - | 4 (0.1) |
| Pathological fracture | 1 (0.1) | - | - | - | - | - | 1 (0.0) |
| Pelvic fracture | 2 (0.1) | - | - | - | - | - | 2 (0.1) |
| Pelvic pain | 1 (0.1) | - | - | - | - | - | 1 (0.0) |
| Peroneal nerve palsy | 1 (0.1) | - | - | 4 (0.4) | - | - | 5 (0.2) |
| Personality change | 1 (0.1) | 1 (1.0) | - | - | - | - | 2 (0.1) |
| Phaeohyphomycosis | 1 (0.1) | - | - | - | - | - | 1 (0.0) |
| Pharyngitis | 1 (0.1) | 1 (1.0) | - | - | - | - | 2 (0.1) |
| Pleomorphism | 1 (0.1) | - | - | - | - | - | 1 (0.0) |
| Pleural effusion | 1 (0.1) | - | - | - | - | - | 1 (0.0) |
| Pneumocystis jirovecii pneumonia | 1 (0.1) | - | - | - | - | - | 1 (0.0) |
| Pneumonia | 3 (0.2) | - | 1 (1.6) | 2 (0.2) | - | - | 6 (0.2) |
| Pneumonia aspiration | 1 (0.1) | - | - | - | - | - | 1 (0.0) |
| Pollakiuria | 2 (0.1) | - | - | - | - | - | 2 (0.1) |
| Poor quality sleep | 1 (0.1) | - | - | - | - | - | 1 (0.0) |
| Post procedural haemorrhage | 1 (0.1) | - | - | - | - | - | 1 (0.0) |
| Posterior cortical atrophy | 1 (0.1) | - | - | - | - | - | 1 (0.0) |
| Precancerous lesion of digestive tract | 1 (0.1) | - | - | - | - | - | 1 (0.0) |
| Prescribed overdose | 1 (0.1) | - | - | - | - | - | 1 (0.0) |
| Prescribed underdose | 10 (0.6) | - | - | 1 (0.1) | - | - | 11 (0.4) |
| Prinzmetal angina | 1 (0.1) | - | - | - | - | - | 1 (0.0) |
| Proctitis | 4 (0.2) | - | - | - | - | - | 4 (0.1) |
| Product dose omission issue | 1 (0.1) | - | - | - | - | - | 1 (0.0) |
| Product substitution issue | 1 (0.1) | - | - | - | - | - | 1 (0.0) |
| Product use in unapproved indication | 8 (0.5) | - | - | - | - | - | 8 (0.3) |
| Progressive multifocal leukoencephalopathy | 14 (0.9) | - | - | 5 (0.5) | - | - | 19 (0.7) |
| Psoriasis | 2 (0.1) | - | - | - | - | - | 2 (0.1) |
| Psychiatric symptom | 1 (0.1) | - | - | 1 (0.1) | - | - | 2 (0.1) |
| Pulmonary histoplasmosis | 1 (0.1) | - | - | - | - | - | 1 (0.0) |
| Pulseless electrical activity | 1 (0.1) | - | - | - | - | - | 1 (0.0) |
| Pyramidal tract syndrome | 1 (0.1) | - | - | - | - | - | 1 (0.0) |
| Pyrexia | 3 (0.2) | - | - | 12 (1.2) | - | - | 15 (0.5) |
| Radiologically isolated syndrome | 2 (0.1) | - | - | - | - | - | 2 (0.1) |
| Radius fracture | 1 (0.1) | - | - | 1 (0.1) | - | - | 2 (0.1) |
| Rash | 3 (0.2) | - | - | - | - | - | 3 (0.1) |
| Rebound effect | 10 (0.6) | - | - | - | - | - | 10 (0.4) |
| Red blood cell count decreased | 1 (0.1) | - | - | 2 (0.2) | - | - | 3 (0.1) |
| Red cell distribution width increased | 1 (0.1) | - | - | - | - | - | 1 (0.0) |
| Reflexes abnormal | 1 (0.1) | - | - | - | - | - | 1 (0.0) |
| Regurgitation | 2 (0.1) | - | - | - | - | - | 2 (0.1) |
| Relapsing-remitting multiple sclerosis | 3 (0.2) | - | - | 1 (0.1) | - | - | 4 (0.1) |
| Relapsing multiple sclerosis | 1 (0.1) | - | - | - | - | - | 1 (0.0) |
| Renal cancer | 1 (0.1) | - | - | 1 (0.1) | - | - | 2 (0.1) |
| Respiratory tract infection | 1 (0.1) | - | - | - | - | - | 1 (0.0) |
| Retroperitoneal disorder | 1 (0.1) | - | - | - | - | - | 1 (0.0) |
| Reversible cerebral vasoconstriction syndrome | 1 (0.1) | - | - | - | - | - | 1 (0.0) |
| Rhinitis | 2 (0.1) | - | - | - | - | - | 2 (0.1) |
| Salpingectomy | 1 (0.1) | - | - | - | - | - | 1 (0.0) |
| SARS-CoV-2 antibody test negative | 1 (0.1) | - | - | - | - | - | 1 (0.0) |
| SARS-CoV-2 test positive | 2 (0.1) | - | - | - | - | - | 2 (0.1) |
| Scoliosis | 1 (0.1) | - | - | - | - | - | 1 (0.0) |
| Secondary progressive multiple sclerosis | 5 (0.3) | - | - | 2 (0.2) | 1 (2.4) | - | 8 (0.3) |
| Sensorimotor disorder | 1 (0.1) | - | - | - | - | - | 1 (0.0) |
| Sensory disturbance | 10 (0.6) | - | - | 1 (0.1) | - | - | 11 (0.4) |
| Sepsis | 1 (0.1) | - | - | 1 (0.1) | - | - | 2 (0.1) |
| Sinusitis | 3 (0.2) | - | - | - | - | - | 3 (0.1) |
| Skeletal injury | 1 (0.1) | - | - | - | - | - | 1 (0.0) |
| Skin burning sensation | 1 (0.1) | - | - | - | - | - | 1 (0.0) |
| Skin cancer | 3 (0.2) | - | - | - | - | - | 3 (0.1) |
| Skin lesion | 5 (0.3) | - | 1 (1.6) | - | - | - | 6 (0.2) |
| Skin reaction | 1 (0.1) | - | - | 1 (0.1) | - | - | 2 (0.1) |
| Somnolence | 1 (0.1) | - | - | 1 (0.1) | - | - | 2 (0.1) |
| Speech disorder | 2 (0.1) | - | - | - | - | - | 2 (0.1) |
| Spinal compression fracture | 1 (0.1) | - | - | - | - | - | 1 (0.0) |
| Spinal cord disorder | 2 (0.1) | - | - | - | - | - | 2 (0.1) |
| Spinal meningeal cyst | 1 (0.1) | - | - | - | - | - | 1 (0.0) |
| Spinocerebellar disorder | 1 (0.1) | - | - | - | - | - | 1 (0.0) |
| Stomatitis | 1 (0.1) | - | - | - | - | - | 1 (0.0) |
| Superficial vein thrombosis | 1 (0.1) | - | - | - | - | - | 1 (0.0) |
| Supranuclear palsy | 1 (0.1) | - | - | - | - | - | 1 (0.0) |
| Syncope | 1 (0.1) | - | - | - | - | - | 1 (0.0) |
| Thalamic infarction | 1 (0.1) | - | - | - | - | - | 1 (0.0) |
| Therapeutic product effect incomplete | 1 (0.1) | - | - | 1 (0.1) | - | - | 2 (0.1) |
| Therapeutic response shortened | 1 (0.1) | - | - | - | - | - | 1 (0.0) |
| Therapy interrupted | 1 (0.1) | - | - | - | - | - | 1 (0.0) |
| Thermal burn | 1 (0.1) | - | - | - | - | - | 1 (0.0) |
| Thrombocytosis | 1 (0.1) | - | - | - | - | - | 1 (0.0) |
| Thyroid disorder | 2 (0.1) | - | - | - | - | - | 2 (0.1) |
| Tinea infection | 1 (0.1) | - | - | - | - | - | 1 (0.0) |
| Tinea pedis | 1 (0.1) | - | - | - | - | - | 1 (0.0) |
| Toxicity to various agents | 1 (0.1) | - | - | - | - | - | 1 (0.0) |
| Tracheal haemorrhage | 1 (0.1) | - | - | - | - | - | 1 (0.0) |
| Transaminases | 1 (0.1) | - | - | - | - | - | 1 (0.0) |
| Transaminases increased | 1 (0.1) | 1 (1.0) | 1 (1.6) | 1 (0.1) | - | - | 4 (0.1) |
| Upper respiratory tract infection | 4 (0.2) | - | - | 1 (0.1) | - | - | 5 (0.2) |
| Urinary incontinence | 1 (0.1) | - | - | 8 (0.8) | - | - | 9 (0.3) |
| Urinary retention | 4 (0.2) | - | - | 1 (0.1) | - | - | 5 (0.2) |
| Urinary tract infection | 9 (0.5) | 3 (2.9) | 1 (1.6) | 9 (0.9) | - | - | 22 (0.8) |
| Uterine cancer | 1 (0.1) | - | - | - | - | - | 1 (0.0) |
| Uveitis | 1 (0.1) | - | - | - | - | - | 1 (0.0) |
| Vaccination failure | 2 (0.1) | - | - | - | - | - | 2 (0.1) |
| Vaccine induced antibody absent | 1 (0.1) | - | - | - | - | - | 1 (0.0) |
| Varicella virus test positive | 1 (0.1) | - | - | - | - | - | 1 (0.0) |
| Varicella zoster virus infection | 1 (0.1) | - | - | - | 1 (2.4) | - | 2 (0.1) |
| Ventricular extrasystoles | 1 (0.1) | - | - | - | - | - | 1 (0.0) |
| Ventricular tachycardia | 1 (0.1) | - | - | 1 (0.1) | - | - | 2 (0.1) |
| Vertigo | 1 (0.1) | - | - | 1 (0.1) | - | - | 2 (0.1) |
| Vestibular disorder | 1 (0.1) | - | - | - | - | - | 1 (0.0) |
| Viral load increased | 1 (0.1) | - | - | - | - | - | 1 (0.0) |
| Viral myocarditis | 1 (0.1) | - | - | - | - | - | 1 (0.0) |
| Viral upper respiratory tract infection | 1 (0.1) | - | - | - | - | - | 1 (0.0) |
| Vision blurred | 4 (0.2) | - | - | 1 (0.1) | - | - | 5 (0.2) |
| Visual acuity reduced | 1 (0.1) | - | - | 1 (0.1) | - | - | 2 (0.1) |
| Visual field defect | 1 (0.1) | - | - | - | - | - | 1 (0.0) |
| Visual impairment | 2 (0.1) | - | - | - | - | - | 2 (0.1) |
| Vitamin D deficiency | 1 (0.1) | - | - | - | - | - | 1 (0.0) |
| Vomiting | 4 (0.2) | - | - | 1 (0.1) | - | - | 5 (0.2) |
| Vulvovaginal warts | 1 (0.1) | - | - | - | - | - | 1 (0.0) |
| Weight increased | 2 (0.1) | - | - | - | - | - | 2 (0.1) |
| White blood cell count decreased | 23 (1.4) | - | 1 (1.6) | 29 (2.9) | - | - | 53 (1.9) |
| White blood cell count increased | 1 (0.1) | - | - | - | - | - | 1 (0.0) |
| Anal cyst | - | - | - | 1 (0.1) | 1 (2.4) | - | 2 (0.1) |
| Arrhythmia | - | - | - | 1 (0.1) | 1 (2.4) | - | 2 (0.1) |
| Blood immunoglobulin G decreased | - | - | - | - | 1 (2.4) | - | 1 (0.0) |
| Loss of consciousness | - | - | - | 2 (0.2) | 1 (2.4) | - | 3 (0.1) |
| Mental impairment | - | - | - | - | 2 (4.8) | - | 2 (0.1) |
| Myelopathy | - | - | - | - | 1 (2.4) | - | 1 (0.0) |
| Product use issue | - | - | - | 2 (0.2) | 1 (2.4) | - | 3 (0.1) |
| Anxiety disorder | - | 1 (1.0) | - | - | - | - | 1 (0.0) |
| Brain oedema | - | 1 (1.0) | - | - | - | - | 1 (0.0) |
| Cerebellar infarction | - | 1 (1.0) | - | - | - | - | 1 (0.0) |
| Colitis microscopic | - | 1 (1.0) | - | - | - | - | 1 (0.0) |
| Dysgeusia | - | 1 (1.0) | - | - | - | - | 1 (0.0) |
| Ear infection | - | 1 (1.0) | - | - | - | - | 1 (0.0) |
| Flushing | - | 1 (1.0) | - | 1 (0.1) | - | - | 2 (0.1) |
| Herpes zoster reactivation | - | 1 (1.0) | - | - | - | - | 1 (0.0) |
| Infusion related reaction | - | 1 (1.0) | - | - | - | - | 1 (0.0) |
| Intentional dose omission | - | 1 (1.0) | - | - | - | - | 1 (0.0) |
| Mental fatigue | - | 1 (1.0) | - | - | - | - | 1 (0.0) |
| Organising pneumonia | - | 1 (1.0) | - | - | - | - | 1 (0.0) |
| Oropharyngeal pain | - | 1 (1.0) | - | 2 (0.2) | - | - | 3 (0.1) |
| Pneumonia staphylococcal | - | 1 (1.0) | - | - | - | - | 1 (0.0) |
| Progressive multiple sclerosis | - | 1 (1.0) | - | - | - | - | 1 (0.0) |
| Renal neoplasm | - | 1 (1.0) | - | - | - | - | 1 (0.0) |
| Ruptured cerebral aneurysm | - | 1 (1.0) | - | - | - | - | 1 (0.0) |
| Staphylococcal bacteraemia | - | 1 (1.0) | - | - | - | - | 1 (0.0) |
| Subarachnoid haemorrhage | - | 1 (1.0) | - | - | - | - | 1 (0.0) |
| Trigeminal neuralgia | - | 1 (1.0) | - | 1 (0.1) | - | - | 2 (0.1) |
| Acne | - | - | 1 (1.6) | - | - | - | 1 (0.0) |
| Erythema | - | - | 2 (3.2) | - | - | - | 2 (0.1) |
| Generalised oedema | - | - | 2 (3.2) | - | - | - | 2 (0.1) |
| Haemorrhoids | - | - | 1 (1.6) | - | - | - | 1 (0.0) |
| Monocyte count increased | - | - | 1 (1.6) | 5 (0.5) | - | - | 6 (0.2) |
| Skin laceration | - | - | 1 (1.6) | - | - | - | 1 (0.0) |
| Trichorrhexis | - | - | 1 (1.6) | - | - | - | 1 (0.0) |
| Unevaluable event | - | - | 1 (1.6) | - | - | - | 1 (0.0) |
| Abdominal symptom | - | - | - | 1 (0.1) | - | - | 1 (0.0) |
| Acute myocardial infarction | - | - | - | 1 (0.1) | - | - | 1 (0.0) |
| Adenocarcinoma pancreas | - | - | - | 1 (0.1) | - | - | 1 (0.0) |
| Adverse event | - | - | - | 1 (0.1) | - | - | 1 (0.0) |
| Affect lability | - | - | - | 1 (0.1) | - | - | 1 (0.0) |
| Aggression | - | - | - | 1 (0.1) | - | - | 1 (0.0) |
| Aortic valve incompetence | - | - | - | 1 (0.1) | - | - | 1 (0.0) |
| Bacterial infection | - | - | - | 1 (0.1) | - | - | 1 (0.0) |
| Blindness cortical | - | - | - | 1 (0.1) | - | - | 1 (0.0) |
| Blood alkaline phosphatase increased | - | - | - | 1 (0.1) | - | - | 1 (0.0) |
| Blood bilirubin increased | - | - | - | 3 (0.3) | - | - | 3 (0.1) |
| Blood creatinine decreased | - | - | - | 2 (0.2) | - | - | 2 (0.1) |
| Blood creatinine increased | - | - | - | 1 (0.1) | - | - | 1 (0.0) |
| Blood urea decreased | - | - | - | 1 (0.1) | - | - | 1 (0.0) |
| Blood urea increased | - | - | - | 1 (0.1) | - | - | 1 (0.0) |
| Burns second degree | - | - | - | 1 (0.1) | - | - | 1 (0.0) |
| Cardiac dysfunction | - | - | - | 1 (0.1) | - | - | 1 (0.0) |
| Cerebral thrombosis | - | - | - | 1 (0.1) | - | - | 1 (0.0) |
| Choking | - | - | - | 3 (0.3) | - | - | 3 (0.1) |
| Cholecystitis | - | - | - | 1 (0.1) | - | - | 1 (0.0) |
| Chromaturia | - | - | - | 1 (0.1) | - | - | 1 (0.0) |
| Clonus | - | - | - | 1 (0.1) | - | - | 1 (0.0) |
| Concomitant disease aggravated | - | - | - | 2 (0.2) | - | - | 2 (0.1) |
| Crohn's disease | - | - | - | 1 (0.1) | - | - | 1 (0.0) |
| Cystitis | - | - | - | 2 (0.2) | - | - | 2 (0.1) |
| Decreased appetite | - | - | - | 1 (0.1) | - | - | 1 (0.0) |
| Drug dose titration not performed | - | - | - | 1 (0.1) | - | - | 1 (0.0) |
| Drug intolerance | - | - | - | 1 (0.1) | - | - | 1 (0.0) |
| Duodenal ulcer | - | - | - | 1 (0.1) | - | - | 1 (0.0) |
| Ecchymosis | - | - | - | 1 (0.1) | - | - | 1 (0.0) |
| Electrocardiogram QT prolonged | - | - | - | 3 (0.3) | - | - | 3 (0.1) |
| Enteritis | - | - | - | 1 (0.1) | - | - | 1 (0.0) |
| Erosive oesophagitis | - | - | - | 1 (0.1) | - | - | 1 (0.0) |
| Erysipelas | - | - | - | 1 (0.1) | - | - | 1 (0.0) |
| Extra dose administered | - | - | - | 2 (0.2) | - | - | 2 (0.1) |
| Facial paresis | - | - | - | 1 (0.1) | - | - | 1 (0.0) |
| Gait spastic | - | - | - | 8 (0.8) | - | - | 8 (0.3) |
| Gastric ulcer | - | - | - | 1 (0.1) | - | - | 1 (0.0) |
| Hallucination, visual | - | - | - | 1 (0.1) | - | - | 1 (0.0) |
| Heart rate decreased | - | - | - | 1 (0.1) | - | - | 1 (0.0) |
| Hepatitis E | - | - | - | 1 (0.1) | - | - | 1 (0.0) |
| Herpes simplex | - | - | - | 1 (0.1) | - | - | 1 (0.0) |
| Hyperaemia | - | - | - | 1 (0.1) | - | - | 1 (0.0) |
| Hyperkinesia | - | - | - | 1 (0.1) | - | - | 1 (0.0) |
| Hypersomnia | - | - | - | 1 (0.1) | - | - | 1 (0.0) |
| Hypertonia | - | - | - | 3 (0.3) | - | - | 3 (0.1) |
| Hypertonic bladder | - | - | - | 3 (0.3) | - | - | 3 (0.1) |
| Hypertransaminasaemia | - | - | - | 3 (0.3) | - | - | 3 (0.1) |
| Hypokinesia | - | - | - | 7 (0.7) | - | - | 7 (0.2) |
| Immature granulocyte percentage increased | - | - | - | 1 (0.1) | - | - | 1 (0.0) |
| Impaired gastric emptying | - | - | - | 1 (0.1) | - | - | 1 (0.0) |
| Incorrect product administration duration | - | - | - | 1 (0.1) | - | - | 1 (0.0) |
| Infestation | - | - | - | 1 (0.1) | - | - | 1 (0.0) |
| Insomnia | - | - | - | 4 (0.4) | - | - | 4 (0.1) |
| Intentional product use issue | - | - | - | 1 (0.1) | - | - | 1 (0.0) |
| Intestinal stenosis | - | - | - | 1 (0.1) | - | - | 1 (0.0) |
| Intracranial aneurysm | - | - | - | 1 (0.1) | - | - | 1 (0.0) |
| Jejunal ulcer | - | - | - | 1 (0.1) | - | - | 1 (0.0) |
| Klebsiella urinary tract infection | - | - | - | 1 (0.1) | - | - | 1 (0.0) |
| Laboratory test abnormal | - | - | - | 1 (0.1) | - | - | 1 (0.0) |
| Lacrimation increased | - | - | - | 1 (0.1) | - | - | 1 (0.0) |
| Lethargy | - | - | - | 1 (0.1) | - | - | 1 (0.0) |
| Leukoplakia oral | - | - | - | 1 (0.1) | - | - | 1 (0.0) |
| Ligament sprain | - | - | - | 1 (0.1) | - | - | 1 (0.0) |
| Lymphoma | - | - | - | 1 (0.1) | - | - | 1 (0.0) |
| Mean cell haemoglobin concentration | - | - | - | 1 (0.1) | - | - | 1 (0.0) |
| Mean cell haemoglobin concentration increased | - | - | - | 1 (0.1) | - | - | 1 (0.0) |
| Mean cell haemoglobin increased | - | - | - | 1 (0.1) | - | - | 1 (0.0) |
| Mean platelet volume increased | - | - | - | 1 (0.1) | - | - | 1 (0.0) |
| Miliaria | - | - | - | 1 (0.1) | - | - | 1 (0.0) |
| Mitral valve incompetence | - | - | - | 1 (0.1) | - | - | 1 (0.0) |
| Mixed anxiety and depressive disorder | - | - | - | 1 (0.1) | - | - | 1 (0.0) |
| Mobility decreased | - | - | - | 3 (0.3) | - | - | 3 (0.1) |
| Mouth ulceration | - | - | - | 1 (0.1) | - | - | 1 (0.0) |
| Muscle atrophy | - | - | - | 1 (0.1) | - | - | 1 (0.0) |
| Muscle rigidity | - | - | - | 1 (0.1) | - | - | 1 (0.0) |
| Muscle tightness | - | - | - | 4 (0.4) | - | - | 4 (0.1) |
| Musculoskeletal discomfort | - | - | - | 7 (0.7) | - | - | 7 (0.2) |
| Musculoskeletal pain | - | - | - | 1 (0.1) | - | - | 1 (0.0) |
| Musculoskeletal stiffness | - | - | - | 2 (0.2) | - | - | 2 (0.1) |
| Mycoplasma test positive | - | - | - | 1 (0.1) | - | - | 1 (0.0) |
| Nail disorder | - | - | - | 1 (0.1) | - | - | 1 (0.0) |
| Neutrophil percentage increased | - | - | - | 1 (0.1) | - | - | 1 (0.0) |
| Noninfective encephalitis | - | - | - | 1 (0.1) | - | - | 1 (0.0) |
| Obstructive sleep apnoea syndrome | - | - | - | 7 (0.7) | - | - | 7 (0.2) |
| Oesophageal ulcer | - | - | - | 1 (0.1) | - | - | 1 (0.0) |
| Onychoclasis | - | - | - | 1 (0.1) | - | - | 1 (0.0) |
| Onycholysis | - | - | - | 1 (0.1) | - | - | 1 (0.0) |
| Onychomycosis | - | - | - | 1 (0.1) | - | - | 1 (0.0) |
| Ophthalmic herpes zoster | - | - | - | 1 (0.1) | - | - | 1 (0.0) |
| Pain in extremity | - | - | - | 1 (0.1) | - | - | 1 (0.0) |
| Pancreatic carcinoma | - | - | - | 1 (0.1) | - | - | 1 (0.0) |
| Pancreatic carcinoma metastatic | - | - | - | 1 (0.1) | - | - | 1 (0.0) |
| Papillary serous endometrial carcinoma | - | - | - | 1 (0.1) | - | - | 1 (0.0) |
| Paralysis | - | - | - | 1 (0.1) | - | - | 1 (0.0) |
| Paraplegia | - | - | - | 1 (0.1) | - | - | 1 (0.0) |
| Periorbital swelling | - | - | - | 1 (0.1) | - | - | 1 (0.0) |
| Peripheral coldness | - | - | - | 1 (0.1) | - | - | 1 (0.0) |
| Peripheral swelling | - | - | - | 2 (0.2) | - | - | 2 (0.1) |
| Photosensitivity reaction | - | - | - | 1 (0.1) | - | - | 1 (0.0) |
| Physical deconditioning | - | - | - | 1 (0.1) | - | - | 1 (0.0) |
| Platelet-large cell ratio decreased | - | - | - | 1 (0.1) | - | - | 1 (0.0) |
| Platelet count decreased | - | - | - | 1 (0.1) | - | - | 1 (0.0) |
| Platelet count increased | - | - | - | 1 (0.1) | - | - | 1 (0.0) |
| Platelet distribution width decreased | - | - | - | 1 (0.1) | - | - | 1 (0.0) |
| Plateletcrit increased | - | - | - | 1 (0.1) | - | - | 1 (0.0) |
| Product size issue | - | - | - | 1 (0.1) | - | - | 1 (0.0) |
| Prostatic disorder | - | - | - | 1 (0.1) | - | - | 1 (0.0) |
| Psychomotor hyperactivity | - | - | - | 1 (0.1) | - | - | 1 (0.0) |
| Psychotic behaviour | - | - | - | 1 (0.1) | - | - | 1 (0.0) |
| Pulmonary fibrosis | - | - | - | 1 (0.1) | - | - | 1 (0.0) |
| Pustule | - | - | - | 1 (0.1) | - | - | 1 (0.0) |
| Pyelonephritis | - | - | - | 1 (0.1) | - | - | 1 (0.0) |
| Quadriparesis | - | - | - | 1 (0.1) | - | - | 1 (0.0) |
| Restlessness | - | - | - | 1 (0.1) | - | - | 1 (0.0) |
| Rheumatoid arthritis | - | - | - | 1 (0.1) | - | - | 1 (0.0) |
| Rhinorrhoea | - | - | - | 2 (0.2) | - | - | 2 (0.1) |
| Seizure | - | - | - | 3 (0.3) | - | - | 3 (0.1) |
| Serum ferritin increased | - | - | - | 1 (0.1) | - | - | 1 (0.0) |
| Shock | - | - | - | 1 (0.1) | - | - | 1 (0.0) |
| Sinus bradycardia | - | - | - | 1 (0.1) | - | - | 1 (0.0) |
| Sinus node dysfunction | - | - | - | 1 (0.1) | - | - | 1 (0.0) |
| Staphylococcal sepsis | - | - | - | 1 (0.1) | - | - | 1 (0.0) |
| Status epilepticus | - | - | - | 1 (0.1) | - | - | 1 (0.0) |
| Stress | - | - | - | 1 (0.1) | - | - | 1 (0.0) |
| Swelling | - | - | - | 1 (0.1) | - | - | 1 (0.0) |
| Swelling of eyelid | - | - | - | 1 (0.1) | - | - | 1 (0.0) |
| Throat clearing | - | - | - | 1 (0.1) | - | - | 1 (0.0) |
| Thrombocytopenia | - | - | - | 1 (0.1) | - | - | 1 (0.0) |
| Thrombosis | - | - | - | 1 (0.1) | - | - | 1 (0.0) |
| Tibia fracture | - | - | - | 1 (0.1) | - | - | 1 (0.0) |
| Tonic convulsion | - | - | - | 1 (0.1) | - | - | 1 (0.0) |
| Tremor | - | - | - | 1 (0.1) | - | - | 1 (0.0) |
| Tricuspid valve incompetence | - | - | - | 1 (0.1) | - | - | 1 (0.0) |
| Tumour marker increased | - | - | - | 1 (0.1) | - | - | 1 (0.0) |
| Underdose | - | - | - | 1 (0.1) | - | - | 1 (0.0) |
| Unresponsive to stimuli | - | - | - | 1 (0.1) | - | - | 1 (0.0) |
| Ureteric cancer | - | - | - | 1 (0.1) | - | - | 1 (0.0) |
| Urinary hesitation | - | - | - | 1 (0.1) | - | - | 1 (0.0) |
| Urinary sediment present | - | - | - | 1 (0.1) | - | - | 1 (0.0) |
| Urine abnormality | - | - | - | 7 (0.7) | - | - | 7 (0.2) |
| Walking disability | - | - | - | 1 (0.1) | - | - | 1 (0.0) |
| Weight decreased | - | - | - | 2 (0.2) | - | - | 2 (0.1) |
| White blood cell disorder | - | - | - | 1 (0.1) | - | - | 1 (0.0) |
| Wrong technique in product usage process | - | - | - | 2 (0.2) | - | - | 2 (0.1) |

Data are expressed as N (%)

**Supplementary Table 4**. Distribution of adverse drug reactions in the High-Level Group Term (HLGTs) of the System Organ Class (SOC) “Nervous system disorders”.

|  | **Fingolimod (N=305)** | **Ozanimod (N=9)** | **Ponesimod (N=2)** | **Siponimod (N=140)** | **Fingolimod/Siponimod (N=15)** | **Overall (N=471)** |
| --- | --- | --- | --- | --- | --- | --- |
| **HLGT** |  |  |  |  |  |  |
| Central nervous system vascular disorders | 8 (2.6) | 3 (33.3) | - | 2 (1.4) | - | 13 (2.8) |
| Cranial nerve disorders (excl neoplasms) | 13 (4.3) | 1 (11.1) | - | 3 (2.1) | - | 17 (3.6) |
| Demyelinating disorders | 86 (28.2) | 1 (11.1) | 1 (50.0) | 27 (19.3) | 4 (26.7) | 119 (25.3) |
| Headaches | 13 (4.3) | - | 1 (50.0) | 11 (7.9) | - | 25 (5.3) |
| Increased intracranial pressure and hydrocephalus | 1 (0.3) | 2 (22.2) | - | - | - | 3 (0.6) |
| Mental impairment disorders | 18 (5.9) | 1 (11.1) | - | 6 (4.3) | 3 (20.0) | 28 (5.9) |
| Movement disorders (incl parkinsonism) | 25 (8.2) | - | - | 31 (22.1) | 1 (6.7) | 57 (12.1) |
| Nervous system neoplasms benign | 1 (0.3) | - | - | - | - | 1 (0.2) |
| Neurological disorders NEC | 126 (41.3) | 1 (11.1) | - | 36 (25.7) | 4 (26.7) | 167 (35.5) |
| Neurological disorders of the eye | 1 (0.3) | - | - | - | - | 1 (0.2) |
| Neuromuscular disorders | 6 (2.0) | - | - | 10 (7.1) | - | 16 (3.4) |
| Peripheral neuropathies | 1 (0.3) | - | - | 4 (2.9) | - | 5 (1.1) |
| Seizures (incl subtypes) | 1 (0.3) | - | - | 8 (5.7) | 1 (6.7) | 10 (2.1) |
| Spinal cord and nerve root disorders | 3 (1.0) | - | - | - | 1 (6.7) | 4 (0.8) |
| Structural brain disorders | 2 (0.7) | - | - | - | 1 (6.7) | 3 (0.6) |
| Central nervous system infections and inflammations | - | - | - | 1 (0.7) | - | 1 (0.2) |
| Sleep disturbances (incl subtypes) | - | - | - | 1 (0.7) | - | 1 (0.2) |

Data are expressed as N (%)

**Supplementary Table 5**. Distribution of adverse drug reactions in the High-Level Group Term (HLGTs) of the System Organ Class (SOC) “Investigations”.

|  | **Fingolimod (N=150)** | **Ozanimod (N=5)** | **Ponesimod (N=7)** | **Siponimod (N=119)** | **Fingolimod/Siponimod (N=3)** | **Overall (N=284)** |
| --- | --- | --- | --- | --- | --- | --- |
| **HLGT** |  |  |  |  |  |  |
| Cardiac and vascular investigations (excl enzyme tests) | 5 (3.3) | - | - | 6 (5.0) | - | 11 (3.9) |
| Cytogenetic investigations | 1 (0.7) | - | - | 1 (0.8) | - | 2 (0.7) |
| Haematology investigations (incl blood groups) | 52 (34.7) | 1 (20.0) | 2 (28.6) | 59 (49.6) | - | 114 (40.1) |
| Hepatobiliary investigations | 50 (33.3) | 4 (80.0) | 5 (71.4) | 35 (29.4) | - | 94 (33.1) |
| Immunology and allergy investigations | 2 (1.3) | - | - | - | 1 (33.3) | 3 (1.1) |
| Investigations, imaging and histopathology procedures NEC | 4 (2.7) | - | - | 1 (0.8) | - | 5 (1.8) |
| Lipid analyses | 1 (0.7) | - | - | - | - | 1 (0.4) |
| Microbiology and serology investigations | 24 (16.0) | - | - | 1 (0.8) | 1 (33.3) | 26 (9.2) |
| Neurological, special senses and psychiatric investigations | 4 (2.7) | - | - | 6 (5.0) | 1 (33.3) | 11 (3.9) |
| Physical examination and organ system status topics | 4 (2.7) | - | - | 2 (1.7) | - | 6 (2.1) |
| Protein and chemistry analyses NEC | 2 (1.3) | - | - | - | - | 2 (0.7) |
| Water, electrolyte and mineral investigations | 1 (0.7) | - | - | 1 (0.8) | - | 2 (0.7) |
| Enzyme investigations NEC | - | - | - | 1 (0.8) | - | 1 (0.4) |
| Renal and urinary tract investigations and urinalyses | - | - | - | 6 (5.0) | - | 6 (2.1) |

Data are expressed as N (%)

**Supplementary Table 6**. Distribution of adverse drug reactions in the High-Level Group Term (HLGTs) of the System Organ Class (SOC) “Infections and infestations”.

|  | **Fingolimod (N=145)** | **Ozanimod (N=11)** | **Ponesimod (N=2)** | **Siponimod (N=61)** | **Fingolimod/Siponimod (N=1)** | **Overall (N=220)** |
| --- | --- | --- | --- | --- | --- | --- |
| **HLGT** |  |  |  |  |  |  |
| Bacterial infectious disorders | 8 (5.5) | 2 (18.2) | - | 5 (8.2) | - | 15 (6.8) |
| Fungal infectious disorders | 36 (24.8) | - | - | 1 (1.6) | - | 37 (16.8) |
| Infections - pathogen unspecified | 44 (30.3) | 5 (45.5) | 2 (100) | 20 (32.8) | - | 71 (32.3) |
| Viral infectious disorders | 57 (39.3) | 4 (36.4) | - | 34 (55.7) | 1 (100) | 96 (43.6) |
| Ectoparasitic disorders | - | - | - | 1 (1.6) | - | 1 (0.5) |

Data are expressed as N (%)

**Supplementary Figure 1.** Reporting odds ratio (ROR) of lymphopenia for each Sphingosine 1-phosphate receptor modulator (S1PRMs) compare to the combination fingolimod/siponimod. CI, confidence interval.


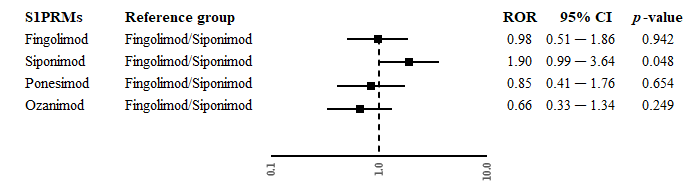

Supplement: Supplementary file 1 — Supplementary Material 1 [file 43440_2025_725_MOESM1_ESM.docx]
